# Entropy Regularization in Deep Reinforcement Learning: A Structured Review Across Classical Control, Generative Policies, and Reasoning Language Models

**DOI:** 10.3390/e28070811

**Published:** 2026-07-16

**Authors:** Giorgio Taricco

**Affiliations:** Department of Electronics and Telecommunications, Politecnico di Torino, Corso Duca degli Abruzzi 24, 10129 Turin, Italy; giorgio.taricco@polito.it

**Keywords:** maximum entropy reinforcement learning, entropy regularization, soft actor–critic, policy mirror descent, trust-region policy optimization, inverse reinforcement learning, offline reinforcement learning, intrinsic motivation, diffusion policies, flow matching, RLVR, reasoning language models, entropy collapse, calibration

## Abstract

Entropy regularization is a recurring mechanism in reinforcement learning (RL), but its meaning changes across algorithmic settings. In classical online RL, entropy encourages exploration and smooths policy improvement; in inverse RL and imitation learning, maximum-entropy resolves ambiguity among expert-consistent behaviors; in offline RL, entropy must be balanced against data support; in generative policies, entropy becomes a tractability problem; and in reinforcement learning with verifiable rewards (RLVR) for large language models (LLMs), token entropy is tied to reasoning diversity, calibration, and collapse. This review organizes these developments into a unified taxonomy. We first summarize the mathematical foundations of maximum-entropy RL, soft Bellman equations, policy-gradient entropy dynamics, and Kullback–Leibler (KL)-constrained mirror descent. We then review entropy in imitation learning, offline RL, intrinsic motivation, diffusion and flow-based policy classes, and RLVR. Particular attention is given to recent work on entropy collapse in reasoning LLMs, entropy-based advantage shaping, covariance-based control, positive-advantage reweighting, and ordinary differential equation (ODE)-based flow-matching policies with tractable entropy. The review emphasizes that entropy is not universally beneficial: useful exploration, support preservation, multimodality, calibration, and reasoning diversity require different entropy objects and different control mechanisms.

## 1. Introduction

Entropy is one of the most persistent ideas in reinforcement learning (RL), but it is not a single operational object. At the level of a stochastic policy, entropy measures uncertainty over actions. At the trajectory level, it measures ambiguity among behavior sequences that may all explain the same reward or demonstration data. At the occupancy measure level, entropy is related to coverage of state–action space. In latent variable and generative policies, entropy is mediated by the sampling process and by the change in variables between latent and action spaces. In token-based language model policies, entropy measures uncertainty over next-token choices, and its collapse can indicate premature concentration on a narrow set of reasoning patterns. These interpretations are mathematically related, but they are not interchangeable. A high-entropy Gaussian actor, a diverse skill repertoire, a conservative offline policy, a diffusion sampler, an ODE-based flow policy, and a reasoning language model with high token entropy can represent very different forms of exploration.

This distinction is central to the present review. Entropy is often introduced as a mechanism for exploration, but in modern deep RL, it also acts as a policy smoother, a trust-region proxy, a likelihood regularizer, a tie-breaker among expert-consistent behaviors, a diversity objective, an uncertainty signal, and a failure diagnostic. The same entropy term can therefore be beneficial in one regime and harmful in another. In online continuous control, entropy regularization encourages stochastic exploration and improves the conditioning of policy improvement through soft Bellman recursions and actor–critic algorithms such as soft Q-learning and Soft Actor–Critic (SAC) [1,2,3]. In trust-region and proximal policy optimization, entropy is often controlled indirectly through KL-constrained updates, clipping, or mirror-descent geometry, as in natural policy gradient, trust-region policy optimization (TRPO), proximal policy optimization (PPO), Maximum a Posteriori Policy Optimization (MPO), and mirror-descent policy optimization [4,5,6,7,8,9,10]. In imitation learning and inverse reinforcement learning (IRL), entropy provides a principled way to select among many expert-consistent policies, thereby resolving behavioral ambiguity rather than merely injecting randomness [1,11,12,13,14]. In offline RL, however, entropy must be balanced against data support: a highly stochastic policy may assign probability to out-of-distribution actions for which learned values are unreliable [15,16,17,18,19].

The need for a unified review has become more urgent because modern policy classes are increasingly expressive. Classical deep RL often relies on Gaussian or categorical policies whose likelihoods and entropies are easy to evaluate. By contrast, diffusion policies, score-based policies, normalizing flows, continuous normalizing flows, and flow-matching policies can represent multimodal action distributions that simple actors cannot, but they change the computational status of entropy. Diffusion policies can generate complex action distributions through iterative denoising, yet their likelihood and entropy are often expensive, approximate, or unavailable during online policy optimization [20,21,22,23,24,25]. Flow-based and ODE-based policies offer a contrasting route: deterministic transport and change in variables formulas can make likelihood and entropy more tractable, although practical estimators still require careful treatment of divergence, numerical integration, and action squashing transformations [26,27,28,29,30]. Thus, the question is no longer only whether entropy should be added to the objective; it is also whether the entropy of the chosen policy class can be computed, estimated without bias, or controlled through a reliable surrogate.

A second recent motivation comes from reinforcement learning for large language models (LLMs), especially reinforcement learning with verifiable rewards (RLVR). In this setting, the policy is a high-dimensional autoregressive distribution over tokens, and entropy is usually measured at the token or response level rather than over continuous actions. PPO-, Group Relative Policy Optimization (GRPO)-, Reinforcement Learning with Leave-One-Out baselines (RLOO)-, and REINFORCE-style updates can rapidly concentrate probability mass on high-reward token sequences. This concentration may improve short-term accuracy while reducing response diversity, worsening calibration, and limiting the model’s ability to explore alternative reasoning paths [31,32,33,34,35,36,37]. Recent work on entropy collapse in reasoning models shows that entropy is no longer merely an auxiliary regularizer; it is an empirical training signal, a diagnostic of policy collapse, and a target for algorithmic intervention.

This review is motivated by three gaps in the current literature. First, classical maximum-entropy RL, control-as-inference, trust-region policy optimization, IRL, offline RL, and intrinsic motivation methods are often reviewed separately even though they share a regularized policy improvement structure. Second, expressive generative policies have changed the meaning of entropy control: a policy can be highly expressive while its entropy is intractable, biased, or only indirectly controlled. Third, LLM post-training has exposed entropy collapse as a practical failure mode whose effects are entangled with calibration, diversity, reasoning depth, and Pass@K performance. These developments require a review that treats entropy not as a single scalar bonus, but as a family of objects whose interpretation depends on the policy class, training regime, and evaluation goal.

The objective of this review is therefore to organize entropy-centered deep RL across these regimes. We ask three guiding questions. First, what object is being regularized or measured: actions, trajectories, occupancies, latent variables, denoising paths, ODE flows, tokens, or complete responses? Second, what mechanism does entropy provide: exploration, robustness, distributional smoothing, ambiguity resolution, support preservation, diversity, calibration, or collapse prevention? Third, when does entropy become a liability: when it slows exploitation, violates dataset support, rewards meaningless randomness, destabilizes likelihood estimation, or encourages low-quality language model outputs? Answering these questions requires connecting results that are usually treated as belonging to separate subfields.

The contribution of this review is organizational and synthetic. We define a taxonomy of entropy objects, mechanisms, and risks; connect classical entropy-regularized RL to trust-region and mirror-descent policy optimization; relate maximum-entropy (MaxEnt) imitation and offline RL to support-aware entropy control; compare entropy tractability across Gaussian, diffusion, flow, and flow-matching policies; and synthesize recent RLVR work on token entropy, entropy collapse, covariance-based explanations, entropy-based advantage shaping, and positive-advantage reweighting. The goal is not to argue that higher entropy is always better. Rather, the goal is to clarify when entropy corresponds to useful exploration, when it simply represents noise, and how algorithm designers can control entropy without obscuring the underlying learning objective.

The paper is organized as follows. Section 1.1 defines the scope and literature taxonomy. Section 2 reviews mathematical foundations of entropy-regularized RL. Section 3, Section 4, Section 5 and Section 6 cover policy-gradient entropy dynamics, trust-region and mirror-descent updates, maximum-entropy imitation learning, offline RL, and diversity-oriented exploration. Section 7 reviews entropy in generative policy classes, including diffusion, score-based, normalizing flow, and flow-matching policies. Section 8 and Section 9 focus on RLVR, entropy collapse in reasoning LLMs, and practical entropy control mechanisms. Section 10 discusses diagnostics and evaluation criteria, Section 11 provides a comparative synthesis and method-selection guidance, and Section 12 concludes with open problems.

### 1.1. Scope and Literature Organization

This review organizes the literature around two axes. The first axis is *what entropy controls*. In classical online RL, entropy usually controls immediate action randomness and encourages exploration. In trajectory-based formulations and IRL, entropy controls ambiguity over complete behaviors. In occupancy measure formulations, entropy is connected to state–action coverage. In offline RL, entropy must be interpreted relative to dataset support, because randomness outside the behavior distribution can amplify extrapolation error. In intrinsic motivation and skill discovery methods, entropy is often replaced or supplemented by diversity and mutual information objectives. In generative policy methods, entropy is tied to latent transport, likelihood evaluation, denoising paths, and change in variables terms. In RLVR for LLMs, entropy controls token-level uncertainty and response-level diversity, but its effects are inseparable from reasoning quality and calibration.

The second axis is *where entropy is measured*. Entropy can be measured over continuous actions, discrete actions, state–action occupancies, latent variables, policy trajectories, diffusion paths, ODE flows, tokens, or complete generated sequences. The measurement choice matters. For example, an entropy bonus on a Gaussian actor has a closed form and directly changes the action distribution. Entropy in a diffusion policy may be inaccessible without approximations. Entropy in a flow policy depends on divergence terms and transformations between latent and action spaces. Token entropy in an LLM can be computed locally, but high token entropy does not necessarily imply useful reasoning exploration. Conversely, low token entropy may indicate confident reasoning, premature collapse, or overfitting, depending on the training stage and evaluation domain.

Table 1 summarizes the resulting taxonomy. The table is intentionally not a chronology. Instead, it is a compact map for reading the remainder of the paper: each later section can be understood as elaborating one row and explaining how entropy changes when the policy class, data regime, or evaluation target changes.

### 1.2. Literature Selection Methodology

To make the scope of the review explicit, we used a structured but non-exhaustive literature selection procedure. We searched arXiv, Google Scholar, IEEE Xplore, ACM Digital Library, and the proceedings of major machine learning and artificial intelligence conferences, including NeurIPS, ICML, ICLR, AAAI, IJCAI, and RSS. The search combined terms related to entropy regularization, maximum-entropy reinforcement learning, policy optimization, actor–critic methods, Soft Actor–Critic, soft Q-learning, imitation learning, inverse reinforcement learning, offline reinforcement learning, generative policies, diffusion policies, flow-matching, RLHF, RLVR, and large language models.

The main time span considered was 2008–2026, beginning with the modern maximum-entropy inverse reinforcement learning formulation in [1]. Earlier information-theoretic references were included when needed for theoretical background. We included works that introduce entropy-based reinforcement learning mechanisms, analyze entropy-regularized objectives, study entropy effects empirically, or use entropy-related criteria in policy optimization, imitation learning, offline RL, generative policies, or LLM post-training. We excluded papers in which entropy appears only as a minor implementation detail, papers without a substantive RL connection, and papers not available in English.

Because several recent LLM/RLVR works are preprints, we distinguish peer-reviewed publications from preliminary technical reports where relevant. Claims based mainly on recent preprints are presented as emerging empirical observations rather than established consensus.

## 2. Mathematical Foundations of Maximum-Entropy RL

**Figure 1:**
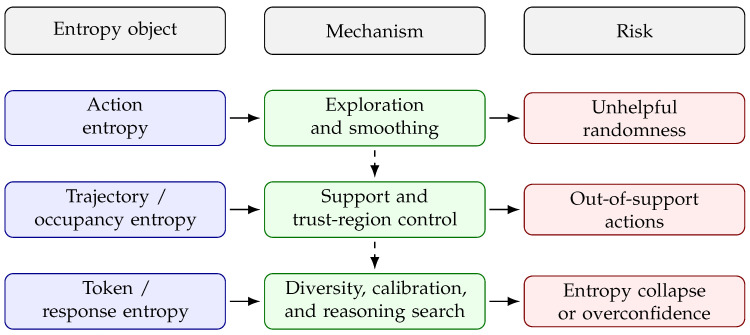
Three-layer interpretation of entropy in deep reinforcement learning. The same entropy value can have different implications depending on whether it is measured over actions, trajectories, occupancies, generated samples, or tokens. Solid lines denote direct methodological links, whereas dashed lines indicate broader conceptual or translational connections between layers.

An RL problem is usually modeled as a Markov decision process $\left(S,A,P,r,\rho_{0},\gamma \right)$, where S is the state space, A is the action space, $P(s^{\prime }|s,a)$ is the transition kernel, r(s,a) is the reward, $\rho_{0}$ is the initial-state distribution, and γ∈[0,1) is the discount factor [38,39,40,41]. For a policy π(a|s), the standard discounted objective is

(1) $$J\left(\pi \right)=E_{\rho_{\pi }}\left[\sum_{t=0}^{\infty }\gamma^{t}r\left(s_{t},a_{t}\right)\right],$$

where $\rho_{\pi }$ denotes the trajectory distribution induced by π, *P*, and $\rho_{0}$. Classical RL therefore searches for a policy that concentrates probability mass on high-return actions. Maximum-entropy RL modifies this criterion by rewarding both task performance and policy uncertainty:

(2) $$J_{\mathrm{ent}}\left(\pi \right)=E_{\rho_{\pi }}\left[\sum_{t=0}^{\infty }\gamma^{t}\left\{r\left(s_{t},a_{t}\right)+\alpha H\left(\pi \left(\cdot |s_{t}\right)\right)\right\}\right],$$

where α>0 controls the reward–entropy trade-off and

(3) $$H\left(\pi \left(\cdot |s\right)\right)=-\int_{A}\pi \left(a|s\right)\log\pi \left(a|s\right)\;da$$

for continuous actions, with the integral replaced by a sum in discrete action spaces. The parameter α is often called the temperature. Large α favors broad action distributions and exploration, while small α recovers the standard reward-maximization objective. The conceptual roots of this formulation include information theory, maximum-entropy inference, linearly solvable control, and path-integral control [42,43,44,45,46,47]. The distinction between entropy objects is summarized visually in Figure 1 and specified more explicitly in Table 2.

Table 2 clarifies the main entropy objects used throughout the review. This distinction is important because an entropy bonus over actions, a KL constraint against a reference policy, an occupancy-diversity objective, and token entropy in an LLM have different mathematical meanings and failure modes.

### 2.1. Generalized Entropies and Their Role in RL

A subtle but important distinction is the precise definition of entropy used in different contexts. The Shannon entropy defined in (3) satisfies four axioms known as the Shannon–Khinchin axioms: continuity, symmetry, maximality, and additivity [48,49]. However, many applications in RL and machine learning use generalizations that relax one or more of these axioms.  

Rényi entropy.  

The Rényi entropy of order α is defined as [50]

(4) $$H_{\alpha }\left(p\right)=\frac{1}{1-\alpha }\log\left(\sum_{i=1}^{n}p_{i}^{\alpha }\right),$$

with α>0,α≠1. As α→1, it recovers Shannon entropy. Rényi entropy is used in RL when the emphasis is on rare events or when the entropy should emphasize different parts of the distribution. Larger α values focus more on high-probability events, while smaller values focus on the support.  

Tsallis entropy.  

Tsallis entropy is defined as [51]

(5) $$H_{q}\left(p\right)=\frac{1}{q-1}\left(1-\sum_{i=1}^{n}p_{i}^{q}\right),$$

where q>0,q≠1. As q→1, it recovers Shannon entropy. Tsallis entropy is non-additive, meaning the entropy of independent systems does not simply add. This property can be useful in RL when rewards or policies are not independent across time steps.  

Cross-entropy and KL divergence.  

The cross-entropy between two distributions *p* and *q* is

(6) $$H\left(p,q\right)=-\sum_{x}p\left(x\right)\log q\left(x\right).$$

Kullback–Leibler (KL) divergence relates cross-entropy and Shannon entropy:

(7) $$D_{\mathrm{KL}}\left(p∥q\right)=H\left(p,q\right)-H\left(p\right).$$

Cross-entropy is frequently used as a loss function in supervised learning and behavior cloning. However, for policy regularization, KL divergence is preferred because it is non-negative, zero only when p=q, and asymmetric (allowing different behavior for mode-seeking vs. mode-covering).  

Implications for RL.  

The choice of entropy measure has practical implications:

- Shannon entropy is most common because it leads to simple gradients and is well-understood theoretically.
- Rényi entropy may be preferred when the policy should preserve rare but important actions.
- Tsallis entropy offers different convergence properties and may handle heavy-tailed distributions.
- Cross-entropy appears in imitation learning, but KL divergence is typically used for trust-region constraints.

Table 3 compares these entropy-like quantities.

This comparison also clarifies why cross-entropy is not the same as entropy regularization. In supervised learning, cross-entropy encourages a predictor to match labels or a target distribution. In RL, the entropy term in (2) instead encourages the policy itself to remain stochastic. KL penalties, by contrast, control the deviation between two policies or between a policy and a reference distribution. The remainder of the review uses “entropy regularization” mainly for Shannon or differential entropy terms added to RL objectives, and uses “KL regularization” when the mechanism is explicitly relative to another distribution.

### 2.2. Soft Bellman Equations and Soft Policy Improvement

Maximum-entropy RL leads to soft Bellman recursions. For a policy π, define the soft state value

(8) $$V^{\pi }\left(s\right)=E_{a∼\pi (\cdot |s)}\left[Q^{\pi }\left(s,a\right)-\alpha \log\pi \left(a|s\right)\right].$$

The corresponding soft Bellman equation is

(9) $$Q^{\pi }\left(s,a\right)=r\left(s,a\right)+\gamma E_{s^{\prime }∼P\left(\cdot |s,a\right)}\left[V^{\pi }\left(s^{\prime }\right)\right].$$

The optimal soft value satisfies

(10) $$V^{⋆}\left(s\right)=\alpha \log\int_{A}\exp\left(Q^{⋆}\left(s,a\right)/\alpha \right)\;da,$$

with a summation replacing the integral in discrete action spaces. The induced optimal policy is

(11) $$\pi^{⋆}\left(a|s\right)=\frac{\exp(Q^{⋆}\left(s,a\right)/\alpha )}{\int_{A}\exp\left(Q^{⋆}\left(\tilde{a}\right)/\alpha \right)\;d\tilde{a}}.$$

These equations show that maximum-entropy RL replaces hard maximization with a log-sum-exp operator. As α→0, the soft value approaches the standard Bellman optimality value. For positive α, the backup accounts for multiple high-value actions rather than only the single maximizer. This smoothing effect is one reason entropy-regularized objectives often improve optimization stability.

Soft policy improvement can be written as a KL projection. Given a soft critic $Q^{\pi }$, the improved policy at state *s* solves

(12) $$\pi_{\mathrm{new}}\left(\cdot |s\right)=\arg\min_{\pi^{\prime }\in \Pi }D_{\mathrm{KL}}\left(\pi^{\prime }\left(\cdot |s\right)∥\frac{\exp(Q^{\pi }\left(s,\cdot \right)/\alpha )}{Z^{\pi }\left(s\right)}\right),$$

where $Z^{\pi }\left(s\right)$ is the normalizing constant. This expression shows that the policy is not simply made random. Rather, it is moved toward a Boltzmann distribution over soft action values. Entropy therefore changes the geometry of policy improvement: policies are updated toward distributions that balance expected value and stochasticity.

### 2.3. Connection to Soft Actor–Critic

Soft Actor–Critic (SAC) is the most influential deep RL algorithm built around the objective (2) [3]. SAC combines off-policy actor–critic learning, clipped double Q-learning, target networks, replay buffers, and stochastic policies trained with soft value. Let $\pi_{\theta }$ be an actor parameterized by θ, let $Q_{\psi }$ be a critic parameterized by ψ, and let D denote the replay buffer. A common actor objective is

(13) $$J_{\pi }\left(\theta \right)=E_{s∼D,a∼\pi_{\theta }\left(\cdot |s\right)}\left[\alpha \log\pi_{\theta }\left(a|s\right)-Q_{\psi }\left(s,a\right)\right].$$

The sign convention in (13) corresponds to minimizing $J_{\pi }\left(\theta \right)$ with respect to θ. Equivalently, the actor maximizes expected soft value $Q_{\psi }\left(s,\;a\right)-\alpha \log\pi_{\theta }\left(a|s\right)$. The entropy term does not merely add noise to actions; it changes the policy improvement target by penalizing overly concentrated distributions.

### 2.4. Temperature Selection and Constrained Entropy

The temperature α determines how aggressively the policy trades reward for entropy. A fixed value can work in simple domains but is often brittle across tasks, action dimensions, and reward scales. Modern maximum-entropy actor–critic methods therefore often adapt α by solving a constrained entropy problem. Instead of maximizing (2) with a fixed coefficient, one may impose a target entropy $\bar{H}$:

(14) $$\max_{\pi }\;E_{\rho_{\pi }}\left[\sum_{t=0}^{\infty }\gamma^{t}r\left(s_{t},\;a_{t}\right)\right]\;s.t.\;E_{s∼d^{\pi }}\left[H\left(\pi \left(\cdot |s\right)\right)\right]\geq \bar{H}.$$

The Lagrangian form introduces α as a dual variable:

(15) $$L\left(\pi ,\alpha \right)=E_{\rho_{\pi }}\left[\sum_{t=0}^{\infty }\gamma^{t}r\left(s_{t},\;a_{t}\right)\right]+\alpha \left(E_{s∼d^{\pi }}\left[H\left(\pi \left(\cdot |s\right)\right)\right]-\bar{H}\right).$$

This perspective clarifies why automatic temperature tuning is more than a heuristic. If the policy entropy is below the target, increasing α places more pressure on stochasticity; if the entropy is above the target, decreasing α permits more exploitation. In continuous control, this mechanism often stabilizes learning. In offline RL or LLM RL, however, a target entropy alone may be insufficient because the relevant constraint is not only how much uncertainty the policy has, but where that uncertainty is placed.

### 2.5. Control as Inference

The control-as-inference view treats optimality as a latent variable and casts policy optimization as probabilistic inference in a graphical model [52,53,54]. A binary optimality variable $O_{t}$ is introduced at each time step, with likelihood

(16) $$p\left(O_{t}=1|s_{t},a_{t}\right)\propto \exp\left(r\left(s_{t},a_{t}\right)/\alpha \right).$$

Conditioning trajectories on optimality gives

(17) $$p\left(\tau |O_{0:T}=1\right)\propto p\left(\tau \right)\exp\left(\frac{1}{\alpha }\sum_{t=0}^{T}r\left(s_{t},a_{t}\right)\right),$$

where p(τ) contains the passive dynamics, initial-state distribution, and any prior policy. Approximate inference in this model yields entropy- or KL-regularized control objectives. In this formulation, rewards act as likelihood factors and regularization terms arise from the divergence between the variational policy and a prior trajectory distribution.

This view is useful because it connects several families of algorithms that may otherwise appear unrelated. Soft Q-learning can be interpreted as learning log-partition functions of an inference problem. KL-regularized policy search can be interpreted as projecting an unnormalized posterior over actions back into a tractable policy class. Imitation learning and IRL can be interpreted as inferring rewards or policies that make expert trajectories probable under a maximum-entropy model. The common structure is a competition between reward likelihood and distributional complexity.

### 2.6. Entropy, KL, and Regularized Policy Improvement

Entropy bonuses are closely related to KL-regularized objectives. A generic regularized improvement step can be written as

(18) $$\pi_{k+1}=\arg\max_{\pi }\;E_{s∼d_{k},a∼\pi }\left[A^{\pi_{k}}\left(s,a\right)\right]-\tau E_{s∼d_{k}}\left[D_{\mathrm{KL}}\left(\pi \left(\cdot |s\right)∥\pi_{k}\left(\cdot |s\right)\right)\right],$$

where $d_{k}$ is a state distribution associated with the current policy and τ>0 controls the strength of the proximity constraint. The solution of the nonparametric version has the advantage-weighted form

(19) $$\pi_{k+1}\left(a|s\right)=\frac{\pi_{k}\left(a|s\right)\exp\left(A^{\pi_{k}}\left(s,a\right)/\tau \right)}{\int_{A}\pi_{k}\left(\tilde{a}|s\right)\exp\left(A^{\pi_{k}}\left(s,\tilde{a}\right)/\tau \right)\;d\tilde{a}}.$$

This expression shows that KL-regularized improvement does not simply maximize entropy. Instead, it reweights the old policy by exponentiated advantage while keeping the new policy close to the old one. The temperature τ plays the role of a trust-region parameter: small τ produces sharper updates toward high-advantage actions, whereas large τ keeps the update closer to the behavior distribution.

Different algorithms arise from different choices of divergence direction, projection family, and approximation strategy. Relative-entropy policy search, natural-gradient methods, TRPO, PPO, MPO, mirror-descent policy optimization, advantage-weighted regression (AWR), and Advantage-Weighted Actor–Critic (AWAC) can all be viewed as instances or approximations of regularized policy improvement [9,10,55,56,57,58]. Forward and reverse KL penalties also have different qualitative effects. A reverse KL term tends to be mode-seeking and may concentrate probability on a subset of high-value actions. A forward KL term tends to be more mode-covering and can preserve broader support. This distinction becomes important in offline RL, where the policy should avoid unsupported actions, and in LLM RL, where token-level updates can collapse probability mass onto a narrow set of responses.

The same structure also explains the connection to recent flow-matching policy optimization. If the improved nonparametric policy in (19) is multimodal, a simple Gaussian actor may be inadequate. A flow-matching actor can instead learn a vector field that transports noise to an advantage-weighted action distribution. FMER uses this idea by interpreting advantage-weighted conditional flow-matching as a simulation-free surrogate for mirror-descent policy improvement while also deriving an entropy correction for bounded actions [30]. This illustrates a recurring theme of the review: entropy is jointly shaped by the objective, the policy update geometry, and the representational capacity of the policy class.

### 2.7. Causal, Trajectory, and Occupancy Entropy

Policy entropy is not the only entropy type used in RL. In sequential decision problems, the entropy of a trajectory distribution

(20) $$H(\tau )=-E_{\tau ∼\rho_{\pi }}\left[\log\;\rho_{\pi }(\tau )\right],$$

contains uncertainty induced by the initial-state distribution, dynamics, and policy. Because the dynamics are usually not controlled by the learner, maximum causal entropy focuses on the policy’s sequential choices rather than on uncontrolled transition noise [1,11]. Informally, causal entropy decomposes policy uncertainty along the trajectory:

(21) $$H_{\mathrm{causal}}\left(\pi \right)=E_{\rho_{\pi }}\left[\sum_{t=0}^{T}H\left(\pi \left(\cdot |s_{t}\right)\right)\right].$$

This is the entropy object most directly connected to maximum-entropy IRL and imitation learning, where the goal is to avoid arbitrary deterministic explanations of expert behavior.

Occupancy measure formulations provide another view. The discounted state–action occupancy measure is

(22) $$d^{\pi }\left(s,a\right)=\left(1-\gamma \right)\sum_{t=0}^{\infty }\gamma^{t}\underset{\pi }{\mathrm{Pr}}\left(s_{t}=s,a_{t}=a\right).$$

Many policy objectives can be written as linear or regularized functions of $d^{\pi }$. Entropy of the occupancy measure encourages coverage of state–action space, while KL constraints between occupancies can express imitation or support matching. This distinction matters because high action entropy at each state does not necessarily imply broad state coverage. A policy can be locally random but remain trapped in a small region of the state space. Conversely, a temporally coherent skill policy may have low instantaneous action entropy but produce diverse trajectories.

### 2.8. Why These Foundations Matter for the Rest of the Review

The mathematical foundations above explain why entropy appears in many apparently different parts of deep RL. In online control, entropy smooths Bellman backups and stabilizes actor–critic learning. In trust-region and mirror-descent methods, KL regularization constrains distributional change and indirectly shapes entropy dynamics. In imitation learning and IRL, causal entropy resolves ambiguity over expert-consistent trajectories. In offline RL, support-aware regularization prevents entropy from pushing the policy toward unreliable actions. In generative policy methods, entropy depends on the likelihood and change in variables properties of the sampler. In LLM RL, token entropy is tractable to compute but difficult to interpret because the relevant uncertainty is tied to reasoning role, response diversity, and calibration.

Thus, the central question is not whether entropy should be maximized. The correct question is which entropy object should be controlled, at which level of the policy, and under which constraints. The remaining sections use this foundation to compare how different research areas answer that question.

## 3. Policy-Gradient Entropy Dynamics and Trust Regions

Policy-gradient algorithms optimize parameters directly, beginning with likelihood-ratio estimators and continuing through actor–critic, natural-gradient, and off-policy variants [4,5,59,60,61,62,63]. Entropy enters these methods either as an explicit bonus or through update constraints. In deep RL, the interaction between entropy and gradient noise is visible in Asynchronous Advantage Actor–Critic (A3C), Deep Q-Network (DQN)-derived systems, deterministic policy methods, Twin Delayed Deep Deterministic Policy-Gradient (TD3), and benchmark studies of reproducibility and hyperparameter sensitivity [64,65,66,67,68,69,70,71].

### 3.1. Entropy Dynamics in Policy Gradients

To understand how entropy changes during policy optimization, consider the standard policy-gradient update with an entropy bonus:

(23) $$\nabla_{\theta }J_{\mathrm{ent}}\left(\theta \right)=E\left[\sum_{t}\nabla_{\theta }\log\pi_{\theta }\left(a_{t}|s_{t}\right)A_{t}\right]+\alpha E\left[\sum_{t}\nabla_{\theta }H\left(\pi_{\theta }\left(\cdot |s_{t}\right)\right)\right].$$

The entropy gradient term has the form

(24) $$\nabla_{\theta }H\left(\pi_{\theta }\left(\cdot |s\right)\right)=-E_{a∼\pi_{\theta }}\left[\left(\log\pi_{\theta }\left(a|s\right)+1\right)\nabla_{\theta }\log\pi_{\theta }\left(a|s\right)\right].$$

This gradient encourages the policy to become more uniform, but its effect is mediated by the likelihood-ratio term. Tokens or actions with low probability receive larger gradient updates from entropy maximization, which can help maintain exploration.

The interaction between entropy and advantage is captured by the variance of the policy gradient:

(25) $$\mathrm{Var}\left[\hat{g}\right]=E\left[{\left(A_{t}\right)}^{2}{\left(\nabla_{\theta }\log\pi_{\theta }\right)}^{2}\right]-{\left(E\left[A_{t}\nabla_{\theta }\log\pi_{\theta }\right]\right)}^{2}.$$

Higher entropy reduces the variance of the likelihood-ratio term, stabilizing learning. However, if the entropy bonus is too large, the policy may remain too stochastic, slowing exploitation.

### 3.2. Natural Gradients, TRPO, and PPO

Natural-gradient methods precondition policy updates by the Fisher information matrix, giving a local geometry for distributional change [4,5,72]. The natural gradient update is

(26) $$\theta_{k+1}=\theta_{k}+\eta F{\left(\theta_{k}\right)}^{-1}\nabla_{\theta }J\left(\theta_{k}\right),$$

where $F\left(\theta \right)=E\left[\nabla_{\theta }\log\pi_{\theta }\nabla_{\theta }\log\pi_{\theta }^{⊤}\right]$ is the Fisher information matrix. For entropy-regularized objectives, the natural gradient has the interpretation of performing mirror-descent in the policy space.

TRPO converts this idea into a constrained optimization problem with a KL trust-region [6]:

(27) $$\max_{\theta }\;E_{s∼d^{\pi_{k}},a∼\pi_{k}}\left[\frac{\pi_{\theta }\left(a|s\right)}{\pi_{k}\left(a|s\right)}A^{\pi_{k}}\left(s,a\right)\right]\;s.t.\;E_{s∼d^{\pi_{k}}}\left[D_{\mathrm{KL}}\left(\pi_{k}\left(\cdot |s\right)∥\pi_{\theta }\left(\cdot |s\right)\right)\right]\leq \delta .$$

The KL constraint indirectly controls entropy by limiting how much the policy distribution can change. If the trust region is too tight, entropy may be preserved but the policy may not improve; if it is too loose, entropy may collapse.

PPO replaces the hard trust-region constraint with clipped likelihood ratios and has become a default in many large-scale RL systems [7,73]. The PPO objective is

(28) $$L^{\mathrm{PPO}}\left(\theta \right)=E\left[\min\left(r_{t}\left(\theta \right)A_{t},\mathrm{clip}\left(r_{t}\left(\theta \right),1-\epsilon ,1+\epsilon \right)A_{t}\right)\right],$$

where $r_{t}\left(\theta \right)=\pi_{\theta }\left(a_{t}|s_{t}\right)/\pi_{\theta_{k}}\left(a_{t}|s_{t}\right)$. The clipping parameter ϵ indirectly affects entropy. Larger ϵ allows larger policy changes, which can lead to faster entropy collapse; smaller ϵ preserves entropy but may slow learning.

### 3.3. Mirror-Descent and Advantage Weighting

Mirror-descent policy optimization, MPO, relative-entropy policy search, AWR, and AWAC update policies toward advantage-weighted action distributions while limiting KL movement [8,9,55,74,75,76]. These algorithms show that entropy control can be expressed as a projection problem: the nonparametric improved policy may be sharp and multimodal, while the parametric actor must approximate it without collapsing prematurely.

The mirror-descent update in policy space takes the form

(29) $$\pi_{k+1}=\arg\min_{\pi }\left[-E_{a∼\pi }A^{\pi_{k}}\left(s,a\right)+\frac{1}{\eta }D_{\mathrm{KL}}\left(\pi ∥\pi_{k}\right)\right],$$

with solution $\pi_{k+1}\left(a|s\right)\propto \pi_{k}\left(a|s\right)\exp\left(\eta A^{\pi_{k}}\left(s,a\right)\right)$. Thus, relative to (19), η=1/τ. The parameter η controls the strength of the advantage tilt rather than adding entropy directly: larger η weakens the KL penalty and usually produces a sharper, more exploitative update when high-advantage actions are concentrated, whereas smaller η keeps the new policy closer to $\pi_{k}$ and therefore tends to preserve the previous stochasticity. Consequently, the entropy change is indirect and depends on both $\pi_{k}$ and the advantage landscape; it should not be interpreted as a monotone entropy-temperature parameter in isolation.

### 3.4. Comparative Analysis of Entropy Control Mechanisms

Different algorithms control entropy through different mechanisms:

- **Entropy bonus (SAC)**: Directly adds entropy to the objective. Most explicit control but requires temperature tuning.
- **KL trust-region (TRPO)**: Indirectly controls entropy through distributional constraints. Preserves diversity but may be conservative.
- **Clipping (PPO)**: Indirectly controls entropy through limiting ratio changes. Trades off exploration and exploitation via ϵ.
- **Mirror-descent (MPO)**: Projects onto advantage-weighted distribution. Provides principled trade-off between exploitation and entropy.
- **Automatic temperature (SAC)**: Adapts entropy bonus to meet target entropy. Most stable in practice.

## 4. Maximum-Entropy Imitation and Inverse Reinforcement Learning

In IRL, entropy answers a different question from its role in online exploration. The learner is not given the reward function directly; instead, it observes expert behavior and must infer a reward or cost under which that behavior is plausible. This problem is inherently underdetermined: many reward functions and many policies can explain the same demonstrations. Entropy therefore acts as an ambiguity resolution principle. Among trajectory distributions that match the expert’s statistics, maximum-entropy inverse reinforcement learning (MaxEnt IRL) selects the least committed distribution, avoiding arbitrary deterministic explanations of expert behavior [1,11].

A standard feature-expectation formulation assumes a trajectory $\tau =(s_{0},a_{0},\ldots ,s_{T},a_{T})$ and a reward parameterization $r_{\theta }\left(\tau \right)=\theta^{⊤}f\left(\tau \right)$, where f(τ) collects trajectory-level feature counts. The MaxEnt IRL distribution takes the form

(30) $$p_{\theta }\left(\tau \right)=\frac{1}{Z(\theta )}\exp\left(r_{\theta }\left(\tau \right)\right),$$

where $Z\left(\theta \right)=\int \exp(r_{\theta }\left(\tau \right))\;d\tau$ is the partition function. The resulting learning problem adjusts θ so that expected feature counts under $p_{\theta }$ match those observed in expert demonstrations. This formulation is maximum-entropy because, subject to the feature-matching constraints, it assigns probability mass as broadly as possible over compatible trajectories. The learned model therefore represents not one expert path, but a distribution over plausible expert-like behaviors.

Maximum causal entropy refines this idea for sequential decision-making by respecting the direction of time. Rather than maximizing entropy over complete trajectories without regard to causality, the causal formulation maximizes the entropy of the action choices conditioned on past states and actions:

(31) $$H_{\mathrm{causal}}\left(\pi \right)=E_{\rho_{\pi }}\left[\sum_{t=0}^{T}H\left(\pi \left(\cdot |s_{t}\right)\right)\right].$$

This distinction matters because the learner should not be credited for uncertainty caused by uncontrolled environment dynamics. The entropy term should reflect uncertainty in the expert’s decisions, not randomness in the transition kernel. In this sense, MaxEnt IRL connects directly to the policy entropy foundations in Section 2, but the purpose changes: entropy no longer exists primarily to discover reward, but to prevent overconfident explanations of demonstrated behavior.

Deep IRL extends these ideas beyond linear reward models and hand-designed features. Guided cost learning replaces fixed feature counts with learned nonlinear cost functions and uses sample-based optimization to fit policies and costs jointly [13]. Adversarial IRL similarly uses discriminator-style objectives to recover rewards from demonstrations while training a policy to match expert behavior [14]. These methods preserve the maximum-entropy logic but move the computational burden from dynamic programming over tabular trajectories to function approximation, sampling, and adversarial training. The entropy principle remains important because deep reward models are expressive enough to overfit demonstrations unless the induced policy distribution is regularized.

Generative Adversarial Imitation Learning (GAIL) reframes imitation as occupancy measure matching with an adversarial discriminator [12]. Instead of explicitly recovering a reward and then solving the resulting RL problem, GAIL trains a policy whose state–action occupancy measure is difficult to distinguish from the expert’s occupancy measure. The objective can be interpreted as matching distributions over visited state–action pairs:

(32) $$d^{\pi }\left(s,a\right)=\left(1-\gamma \right)\sum_{t=0}^{\infty }\gamma^{t}{\mathrm{Pr}}_{\pi }\left(s_{t}=s,a_{t}=a\right).$$

From the entropy perspective, this is a shift from trajectory probability modeling to occupancy matching. The policy is not merely asked to choose expert-like actions at individual expert states; it is asked to induce a distribution over state–action visitation that resembles the expert’s. This occupancy view clarifies why imitation learning is sensitive to compounding errors: a small action mismatch can move the learner into states that are absent from the demonstrations, after which supervised action matching provides little guidance.

Behavioral cloning occupies the opposite end of the spectrum. It treats imitation as supervised learning and directly maximizes the likelihood of expert actions under $\pi_{\theta }\left(a|s\right)$. This is simple and scalable, but it ignores the learner-induced state distribution. Dataset Aggregation (DAgger) addresses this distribution shift problem by iteratively collecting expert labels on states visited by the learner [77]. Surveys of imitation learning emphasize that this covariate shift issue is central: matching expert actions on expert states does not guarantee robust behavior under learner-induced states [78]. Entropy can partially mitigate the problem by keeping multiple plausible actions available, but it cannot substitute for coverage of relevant states.

Advantage-weighted imitation and related methods connect imitation back to regularized policy improvement [75]. Rather than cloning all demonstrated actions equally, these methods weight actions according to estimated value or advantage. This creates a bridge between imitation, offline RL, and mirror-descent policy optimization: the learner remains close to demonstration data while emphasizing actions that appear more useful. Entropy and KL regularization are important in this setting because they determine whether the policy preserves behavioral support or collapses onto a narrow subset of high-weight samples. A highly concentrated imitation policy may achieve low training error but fail when expert behavior is multimodal or when several distinct strategies solve the same task.

The main lesson is that entropy in imitation and IRL is not simply exploration noise. It is a modeling assumption about ambiguity. Expert demonstrations rarely identify a unique policy or reward. Maximum-entropy formulations acknowledge this non-identifiability by preferring broad distributions consistent with the evidence. Adversarial imitation and occupancy matching extend the idea to high-dimensional deep policies, while behavioral cloning and DAgger reveal the practical consequences of distribution shift. The same tension will reappear in offline RL: entropy is useful when it preserves plausible alternatives inside the data distribution, but harmful when it assigns probability to unsupported actions.

## 5. Offline RL, Conservative Objectives, and Support-Aware Entropy

Offline RL optimizes a policy from a fixed dataset $D={\left\{\left(s_{i},a_{i},r_{i},s_{i}^{\prime }\right)\right\}}_{i=1}^{N}$, without additional interaction with the environment. This changes the role of entropy. In online RL, stochasticity can help the agent discover new rewarding states and correct poor value estimates through additional data collection. In offline RL, the policy cannot freely explore: actions outside the dataset support may have poorly estimated values, and exploiting these errors can produce policies that look good under the learned critic but fail when deployed [15,79,80]. Entropy is therefore useful only when it preserves uncertainty inside reliable support. A high-entropy policy that spreads probability mass over unsupported actions is not exploratory in the useful online sense; it is extrapolating beyond the data.

This support issue can be stated in terms of the behavior distribution. Let $\pi_{\beta }\left(a|s\right)$ denote the policy, explicit or implicit, that generated the offline dataset. If π(a|s) assigns substantial probability to actions for which $\pi_{\beta }\left(a|s\right)$ is small or zero, then the learned value Q(s,a) must extrapolate. A support-aware offline objective therefore often combines policy improvement with a constraint such as

(33) $$E_{s∼D}\left[D_{\mathrm{KL}}\left(\pi \left(\cdot |s\right)∥\pi_{\beta }\left(\cdot |s\right)\right)\right]\leq \epsilon ,$$

or with another divergence, distance, or implicit regularizer that discourages large deviation from dataset actions. The precise divergence matters. A reverse-KL-like constraint can be mode-seeking and may select one high-value behavior mode from the dataset, whereas a forward-KL-like or maximum-likelihood constraint can be more mode-covering. Thus, entropy in offline RL is inseparable from support: the objective must decide not only how stochastic the learned policy should be, but also where that stochasticity is allowed.

Behavior-regularized methods make this principle explicit. Batch-Constrained deep Q-learning (BCQ) restricts candidate actions to those likely under a generative model trained on the dataset, thereby avoiding arbitrary maximization over unsupported actions [19]. Bootstrapping Error Accumulation Reduction (BEAR) constrains the learned policy to remain close to the behavior policy using a distributional discrepancy [81]. Behavior-Regularized Actor–Critic (BRAC) studies offline actor–critic objectives with explicit behavior-policy regularization [18]. In these methods, entropy is not simply added as an independent exploration bonus. A stochastic actor is acceptable only to the extent that its probability mass remains within the empirical action support.

Conservative value learning methods address the same pathology from the critic side. Conservative Q-Learning (CQL) penalizes high values assigned to actions not well supported by the dataset, reducing the incentive for the policy to exploit extrapolation error [16]. A stylized form of the conservative penalty is

(34) $$L_{\mathrm{CQL}}\left(Q\right)=L_{\mathrm{Bellman}}\left(Q\right)+\lambda \left(E_{s∼D,a∼\mu (\cdot |s)}\left[Q\left(s,a\right)\right]-E_{s∼D,a∼D}\left[Q\left(s,a\right)\right]\right),$$

where μ is a proposal distribution over actions. The penalty lowers values for sampled actions outside the dataset relative to observed actions. From the entropy perspective, this changes the policy improvement landscape: even if the actor is stochastic, unsupported actions should not appear artificially attractive. Conservative critics therefore make entropy safer by reshaping the value function rather than by directly constraining the actor entropy.

Implicit Q-Learning (IQL) takes another route by avoiding explicit maximization over out-of-distribution actions [17]. It learns a value function through expectile regression and extracts a policy by advantage-weighted regression on dataset actions. A representative extraction objective is

(35) $$\max_{\pi }\;E_{(s,a)∼D}\left[\exp(A(s,a)/\tau )\log\pi (a|s)\right],$$

where A(s,a) is an estimated advantage and τ controls the sharpness of advantage weighting. Advantage-Weighted Actor–Critic (AWAC) uses a related idea, weighting behavior actions by estimated advantage while remaining anchored to the dataset [76]. These methods connect offline RL to mirror-descent and imitation learning: policy improvement is performed by reweighting observed actions rather than by freely optimizing over the full action space. Entropy is implicitly governed by the diversity of high-advantage dataset actions and by the temperature of the advantage weights.

Model-based offline RL adds a further source of uncertainty. Instead of learning only a value function or policy from D, model-based methods learn a dynamics model and use it for rollout, planning, or trajectory optimization. This can improve data efficiency, but model errors compound when the planner visits states or actions outside the dataset. Model-based Offline Policy Optimization (MOPO) penalizes model uncertainty during synthetic rollouts, while Conservative Offline Model-Based Policy Optimization (COMBO) combines conservative value learning with model-based data generation [82,83]. Trajectory optimization methods such as Trajectory Transformer and model-based planning with sequence models similarly rely on learned generative models of behavior [84,85]. In this setting, entropy appears both in the policy and in the learned model or trajectory sampler. A diverse planner is useful only if its generated trajectories remain plausible under the offline data distribution.

Sequence-modeling approaches reinterpret offline RL as conditional generation over trajectories. Decision Transformer models trajectories autoregressively and conditions generation on desired return, converting policy learning into sequence prediction [86]. Trajectory Transformer models state–action–reward sequences and uses planning or beam search over generated continuations [84]. Diffuser and Decision Diffuser use diffusion-style generative modeling to sample trajectories or plans conditioned on goals, returns, or constraints [21,85]. Implicit Diffusion Q-Learning and related approaches combine expressive generative policies with value guidance [87]. In these methods, entropy is no longer only a scalar actor bonus; it is a property of the generative sequence distribution, the conditioning variable, the sampler, and the decoding or planning procedure.

This generative view exposes a subtle distinction. A high-entropy trajectory model may represent many plausible futures from the dataset, which is beneficial for planning under ambiguity. However, high entropy in the decoding process can also produce incoherent or low-value trajectories. Conversely, aggressive conditioning on high return can collapse the generative distribution to a narrow set of trajectories, improving benchmark score while reducing robustness. Thus, offline sequence models face a version of the same entropy trade-off seen in LLM RL: diversity is useful only when it corresponds to meaningful alternatives rather than arbitrary samples.

Offline RL therefore reverses the naive maximum-entropy lesson. In online RL, entropy is often valuable because new data can verify or correct exploratory actions. In offline RL, entropy must be coupled to support constraints, conservative value estimation, or generative plausibility. The central question is not whether the policy has high entropy, but whether its uncertainty is concentrated on actions and trajectories for which the dataset provides evidence. This support-aware interpretation links offline RL to imitation learning on one side and to generative policy classes on the other: all three settings require distinguishing useful distributional breadth from unsupported randomness.

## 6. Intrinsic Motivation, Diversity, and Skill Discovery

A second branch of entropy-centered RL generalizes entropy from immediate action randomness to behavioral diversity. In this view, the central problem is not only how to make a policy stochastic at a given state, but how to induce broad, informative, and reusable experience over time. A policy can have high action entropy while still visiting a small portion of the state space; conversely, a temporally coherent skill may have low instantaneous action entropy but produce diverse and useful trajectories. This distinction motivates exploration objectives based on uncertainty, novelty, information gain, state coverage, controllability, and mutual information.

Classical exploration theory provides several foundations for this view. Count-based exploration rewards rarely visited states or state–action pairs, optimism under uncertainty assigns high value to insufficiently explored regions, posterior sampling maintains uncertainty over models or value functions, and bandit theory formalizes exploration–exploitation trade-offs through regret bounds [88,89,90,91,92,93,94,95]. These methods are not always described as entropy regularization, but they address a related objective: preventing the learner from collapsing too early onto a narrow behavioral distribution. The difference is that classical uncertainty-driven methods often direct exploration toward epistemically uncertain regions, whereas a raw entropy bonus may increase randomness without regard to whether the resulting data are informative.

Deep RL made this distinction more important because large state spaces make exact counts and tabular uncertainty estimates impractical. Curiosity-driven methods reward prediction error or learning progress, encouraging the agent to visit transitions that are difficult for its internal model to predict [96]. Random Network Distillation (RND) uses prediction error against a fixed random target network as a novelty signal [97]. Episodic novelty methods compare current observations to an episodic memory, encouraging the agent to avoid repeatedly visiting the same states within an episode. Go-Explore-style archive expansion separates the problem of reaching diverse states from the problem of robustly solving the task, using an archive of promising states or trajectories to drive systematic exploration [98]. Agent57 and related large-scale agents combine intrinsic rewards, memory, and distributed training to balance short-term exploitation with long-horizon exploration [99,100]. Across these methods, entropy is no longer merely a local property of π(a|s); it becomes a proxy for the diversity and novelty of the data distribution induced by the policy.

A useful way to formalize this shift is through state or occupancy coverage. The discounted occupancy measure

(36) $$d^{\pi }\left(s,a\right)=\left(1-\gamma \right)\sum_{t=0}^{\infty }\gamma^{t}\underset{\pi }{\mathrm{Pr}}\left(s_{t}=s,a_{t}=a\right)$$

captures where the policy actually goes, not only how random its action choices are. Objectives that encourage high entropy in $d^{\pi }$ promote broad state–action visitation:

(37) $$H\left(d^{\pi }\right)=-\int d^{\pi }\left(s,a\right)\log d^{\pi }\left(s,a\right)\;ds\;da.$$

This is a stronger and more global requirement than maximizing H(π(·|s)) at each visited state. A policy may randomize among actions that all keep it in the same region, yielding high local entropy but poor coverage. Conversely, structured exploration may require committing to temporally extended actions that reduce local entropy while increasing trajectory-level diversity.

Unsupervised skill discovery methods make this idea explicit by learning a family of policies indexed by a latent skill variable *z*. The goal is to produce behaviors that are distinguishable, diverse, and reusable before a downstream reward is specified. Variational Intrinsic Control and related methods use mutual information between latent skills and states or trajectories to encourage the agent to discover controllable behavioral modes [101,102]. Diversity Is All You Need (DIAYN) maximizes a mutual information objective between skills and visited states while also encouraging high-entropy action choices within each skill [103]. A representative objective is

(38) $$\max_{\pi ,q}\;I\left(z;s\right)-\beta I\left(a;s|z\right)$$

or, in a common variational form

(39) $$\max_{\pi ,q}\;E_{z∼p\left(z\right),s∼d^{\pi_{z}}}\left[\log q\left(z|s\right)-\log p\left(z\right)\right]+\alpha E_{s∼d^{\pi_{z}}}\left[H\left(\pi_{z}\left(\cdot |s\right)\right)\right],$$

where q(z|s) is a discriminator that predicts the skill from the visited state. The mutual information term encourages different skills to visit distinguishable states, while the policy entropy term prevents each skill from becoming prematurely deterministic. This separation is important: diversity across skills and entropy within a skill are different objects.

Empowerment and controllability objectives provide another route to entropy-like exploration. Empowerment measures how much influence an agent’s actions have over future states, often through mutual information between actions or skills and future observations [104,105,106]. Rather than rewarding unpredictability itself, empowerment rewards the agent for reaching states from which it has many reliable options. This is closer to controllable diversity than to random behavior. A noisy policy in an uncontrollable region may have high action entropy but low empowerment, whereas a policy that reaches a bottleneck state with many possible futures may have high strategic value even if its actions are locally structured.

These methods reveal a key limitation of raw policy entropy: random actions are not equivalent to diverse, controllable, reusable skills. In sparse-reward domains, entropy bonuses can help early exploration, but they often fail when meaningful progress requires coordinated behavior over long horizons. Intrinsic motivation and skill discovery address this limitation by moving the entropy object from single-step action uncertainty to state coverage, trajectory diversity, information gain, and controllability. This broader interpretation also connects to offline and generative RL. Offline datasets with diverse behavior support stronger policy improvement; generative trajectory models depend on meaningful diversity in the data distribution; and LLM reasoning benefits from diverse solution paths only when they correspond to useful alternatives rather than arbitrary variation.

The practical challenge is to make diversity task-relevant. Curiosity based purely on prediction error can be distracted by stochastic but uncontrollable observations. State-coverage objectives can reward irrelevant regions of the environment. Skill discovery objectives can produce behaviors that are diverse but not useful for downstream tasks. Thus, entropy and diversity objectives require an additional notion of usefulness: novelty should be informative, skills should be controllable, and behavioral diversity should improve transfer or robustness. This same issue reappears in RLVR for language models, where high token entropy is useful only when it preserves meaningful reasoning alternatives rather than surface-level randomness.

### Illustrative Numerical Example: Local Entropy Can Reduce Useful Diversity

The distinction between local entropy and useful diversity can be illustrated by a small finite-horizon MDP. The purpose of the example is not to benchmark an algorithm, but to isolate a failure mode: maximizing local action entropy can increase $E[H\left(\pi \left(\cdot |s_{t}\right)\right)]$ while reducing return, state coverage, and useful branch exploration.

The MDP has five states. The initial state $s_{0}$ has two actions. Action $a_{0}$ enters a noisy low-reward branch $s_{N}$, while action $a_{1}$ enters a useful branch $s_{U}$. In $s_{N}$, three actions are available and all self-loop with reward 0.1. In $s_{U}$, action $b_{0}$ reaches a successful terminal state with reward 1, while action $b_{1}$ reaches a failure terminal state with reward 0. The horizon is H=4. We compare three fixed policies chosen to represent different qualitative update outcomes:

(40) $$\begin{array}{cccc} \mathrm{Policy} & \pi (\cdot |s_{0}) & \pi (\cdot |s_{N}) & \pi (\cdot |s_{U})\\ ine\mathrm{Reward}\;\mathrm{only} & \left(0.05,0.95\right) & \left(1/3,1/3,1/3\right) & \left(0.95,0.05\right)\\ \mathrm{Reward}\;+\;\mathrm{action}\;\mathrm{entropy} & \left(0.80,0.20\right) & \left(1/3,1/3,1/3\right) & \left(0.50,0.50\right)\\ \mathrm{Reward}\;+\;\mathrm{coverage}\;\mathrm{bonus} & \left(0.35,0.65\right) & \left(1/3,1/3,1/3\right) & \left(0.90,0.10\right) \end{array}$$

The second policy has the largest local stochasticity, but it assigns most probability to the noisy self-looping branch. Thus, its entropy is high for the wrong reason.

For each policy, all trajectories are enumerated exactly. The average local action entropy is computed over executed nonterminal decisions:

(41) $${\bar{H}}_{\mathrm{act}}\left(\pi \right)=\frac{E_{\pi }\left[\sum_{t=0}^{H-1}1\left\{s_{t}\notin T\right\}H\left(\pi \left(\cdot |s_{t}\right)\right)\right]}{E_{\pi }\left[\sum_{t=0}^{H-1}1\left\{s_{t}\notin T\right\}\right]},$$

where T is the set of terminal states. State coverage is measured as the expected number of distinct states visited in a trajectory. The exact enumeration procedure used to compute these quantities is summarized in Algorithm 1.
**Algorithm 1** Exact enumeration for the toy entropy MDP.**Require:** Policy π, horizon *H*, transition map *P*, reward map *r*  1:Initialize accumulators for return, entropy sum, decision count, coverage, useful-branch probability, and success probability  2:Enumerate all trajectories $\tau =(s_{0},a_{0},\ldots ,s_{H})$ induced by π  3:**for** each trajectory τ **do**  4:    Compute trajectory probability $p\left(\tau \right)=\prod_{t}\pi \left(a_{t}|s_{t}\right)$  5:    Compute return $G\left(\tau \right)=\sum_{t}r\left(s_{t},a_{t}\right)$  6:    Compute entropy contribution $\sum_{t}1\left\{s_{t}\notin T\right\}H\left(\pi \left(\cdot |s_{t}\right)\right)$  7:    Compute number of nonterminal decisions and number of distinct visited states  8:    Add p(τ)-weighted quantities to the accumulators  9:**end for**10:Normalize the entropy accumulator by the expected number of nonterminal decisions11:**return** Expected return, average local entropy, expected coverage, useful-branch probability, and success probability

Table 4 reports the resulting metrics and shows that the largest local action entropy corresponds to the lowest return and success probability. The code used for this exact enumeration is provided in Appendix A.

The surprising effect is that the policy with the largest local action entropy performs worst. It spends most of its probability mass in the noisy self-looping branch, where entropy is high but behavior is not useful. The reward-only policy has much lower local entropy, but it reaches the useful branch with high probability and obtains the largest return. The coverage-oriented policy is intermediate: it preserves more diversity than the reward-only policy while avoiding the most severe failure of the local entropy-biased policy.

This toy construction reinforces the main point of the section. Local action entropy, occupancy coverage, trajectory diversity, and useful exploration are different objects. A scalar entropy bonus can increase randomness without increasing controllable or reward-relevant diversity. This motivates the transition to generative policy classes: richer policies can represent diverse behaviors, but their entropy must still be interpreted through the sampler and the induced trajectory distribution rather than through a local entropy statistic alone.

## 7. Entropy in Generative Policy Classes

The illustrative MDP in the previous section shows that local action entropy can increase while useful trajectory diversity decreases. Generative policy classes address a related but distinct problem: they make it possible to represent richer action or trajectory distributions, including multimodal behaviors that simple Gaussian actors cannot capture. However, expressiveness alone does not determine whether entropy is useful. A generative actor may produce diverse samples, but the relevant questions are whether its likelihood is tractable, whether its entropy can be estimated, and whether its diversity corresponds to reward-relevant behavior rather than unsupported or noisy variation.

Gaussian and categorical policies are often too restrictive for multimodal action landscapes. In continuous control, a diagonal Gaussian actor can represent local uncertainty but cannot easily model separated action modes, contact-rich behaviors, or multiple valid solutions to the same state. In offline RL and imitation learning, this limitation is especially severe because the dataset may contain several distinct expert or near-expert behaviors. Collapsing these behaviors into a unimodal policy can produce unrealistic averaged actions. Generative policy classes therefore improve representational capacity, but they also change the computational status of entropy.

The key question is whether the policy permits tractable sampling, likelihood evaluation, and entropy estimation at the same time. Classical Gaussian policies satisfy all three requirements but have limited expressiveness. Energy-based policies can represent complex distributions, but their normalizing constants are usually intractable. Diffusion policies and score-based policies provide highly expressive sampling mechanisms, yet exact likelihoods and entropies are often expensive or unavailable during policy optimization. Normalizing flows, continuous normalizing flows (CNFs), and flow-matching policies offer a middle ground: they can represent complex action distributions through learned transformations while retaining a connection to likelihood and entropy through change in variables formulas [26,27,28,29,107,108,109].

For an invertible transformation $a=f_{\theta }\left(z;s\right)$ with latent variable $z∼p_{0}\left(z\right)$, the policy density is

(42) $$\log\pi_{\theta }\left(a|s\right)=\log p_{0}\left(z\right)-\log\left|\det\frac{\partial f_{\theta }\left(z;s\right)}{\partial z}\right|,\;z=f_{\theta }^{-1}\left(a;s\right).$$

The corresponding entropy satisfies

(43) $$H\left(\pi_{\theta }\left(\cdot |s\right)\right)=H\left(p_{0}\right)+E_{z∼p_{0}}\left[\log\left|\det\frac{\partial f_{\theta }\left(z;s\right)}{\partial z}\right|\right].$$

Thus, entropy is controlled by both the base distribution and the volume change induced by the transformation. This is fundamentally different from a Gaussian actor, where entropy is determined by the covariance alone. In a flow policy, entropy can vary across state-dependent nonlinear deformations, allowing the actor to represent multimodality and anisotropic uncertainty more flexibly.

Continuous normalizing flows replace a discrete sequence of invertible transformations with an ODE:

(44) $$\frac{dz_{t}}{dt}=v_{\theta }\left(z_{t},t,s\right),\;z_{0}∼p_{0},\;a=z_{1}.$$

The log density evolves according to the instantaneous change in variables equation

(45) $$\frac{d}{dt}\log p_{t}\left(z_{t}|s\right)=-\mathrm{tr}\left(\frac{\partial v_{\theta }\left(z_{t},t,s\right)}{\partial z_{t}}\right).$$

Equivalently, the entropy changes with the expected divergence of the velocity field:

(46) $$H\left(p_{1}\right)=H\left(p_{0}\right)+E\left[\int_{0}^{1}\mathrm{tr}\left(\frac{\partial v_{\theta }\left(z_{t},t,s\right)}{\partial z_{t}}\right)\;dt\right].$$

This makes ODE-based policies attractive for entropy-aware RL because the same vector field that transports samples also determines likelihood and entropy. The difficulty is computational: evaluating the divergence term can be expensive in high dimensions, and numerical integration introduces solver error, cost, and possible bias. Hutchinson-style trace estimators and FFJORD-like methods make this practical but do not eliminate the variance and implementation complexity [26,27].

Flow-matching learns such vector fields by regressing to target velocities along probability paths between a simple base distribution and a complex data distribution [28,29,108,109]. Unlike diffusion training, flow-matching can be simulation-free during training: the model learns the velocity field from sampled intermediate states rather than by explicitly simulating a reverse stochastic chain. For policy learning, this is useful because the actor can be trained to transport latent noise to actions that match demonstrations, high-value samples, or advantage-weighted targets. The resulting policy can remain expressive while preserving a tractable route to likelihood and entropy through ODE transport.

### 7.1. Diffusion Policies

Diffusion models generate samples through iterative denoising and have become important in vision, planning, and behavior modeling [20,21,22,110,111,112,113,114,115]. A diffusion model defines a forward noising process that gradually maps data to noise and learns a reverse process that transforms noise back into data. In policy learning, the generated object may be a single action, an action sequence, a trajectory segment, or a complete plan. This makes diffusion policies attractive in domains where the conditional action distribution is multimodal.

In RL, diffusion policies and diffusion-based offline RL exploit this expressive sampling process to represent multimodal expert or dataset action distributions [23,24,25,116,117,118]. In imitation learning and offline RL, diffusion policies can avoid the averaging problem of Gaussian behavioral cloning. Given a state *s*, a diffusion actor can sample several distinct plausible actions rather than forcing them into one unimodal density. In planning, diffusion models can generate trajectory candidates that satisfy constraints or match return-conditioning signals. In value-guided variants, a critic or Q-function can bias denoising toward higher-return samples.

The entropy problem is computational. A diffusion sampler defines a complex distribution through many denoising steps, but the exact marginal likelihood $\pi_{\theta }\left(a|s\right)$ is usually not available in a simple closed form. Entropy is even harder: estimating

(47) $$H\left(\pi_{\theta }\left(\cdot |s\right)\right)=-E_{a∼\pi_{\theta }\left(\cdot |s\right)}\left[\log\pi_{\theta }\left(a|s\right)\right]$$

requires access to the policy density or to an accurate surrogate. As a result, many diffusion policy methods avoid direct entropy maximization and instead use regression losses, denoising objectives, critic guidance, behavior cloning terms, or approximate likelihood surrogates. These methods may still preserve behavioral diversity, but the mechanism is not the same as an explicit entropy bonus in SAC.

This distinction matters for policy optimization. In actor–critic RL, a tractable log probability allows likelihood-ratio gradients, KL penalties, entropy bonuses, and trust-region constraints. If the actor is a diffusion sampler with intractable likelihood, these tools become harder to apply directly. Some methods therefore treat the diffusion model as a behavior prior and use value guidance at sampling time; others train the diffusion policy toward high-value actions generated from the dataset or from a critic. In both cases, entropy is controlled indirectly by the denoising noise schedule, sampling temperature, guidance strength, number of denoising steps, and the diversity of the training data.

Diffusion policies also expose a difference between sample diversity and policy entropy. A sampler may generate diverse actions because the reverse process is stochastic, but this does not automatically imply a calibrated or controllable action density. Strong critic guidance can improve return while reducing diversity; weak guidance can preserve diversity while generating low-value actions. The same trade-off appears in sequence-level diffusion planners: broad trajectory generation improves coverage, but unconstrained sampling can leave the data manifold. Thus, diffusion policies shift entropy control from a simple analytic term to a design problem involving the sampler, guidance mechanism, and training distribution.

### 7.2. Flow-Matching and ODE-Based Entropy

Flow-matching policies offer a contrasting design. By learning a deterministic vector field and sampling through ODE integration, they can preserve expressive multimodality while making likelihood and entropy more tractable. The policy is represented by transporting a base random variable $z_{0}∼p_{0}$ to an action $a=z_{1}$ according to

(48) $$\frac{dz_{t}}{dt}=v_{\theta }\left(z_{t},t,s\right),\;t\in \left[0,1\right].$$

When the ODE map is well defined and invertible, density and entropy can be tracked through the divergence of $v_{\theta }$. This provides a direct connection between generative modeling and entropy-aware policy optimization.

In an RL setting, the target action distribution is not merely the empirical behavior distribution. Policy improvement should favor actions with high advantage while retaining enough support to avoid collapse. A mirror-descent update suggests a nonparametric target of the form

(49) $$q_{k}\left(a|s\right)\propto \pi_{k}\left(a|s\right)\exp\left(A^{\pi_{k}}\left(s,a\right)/\tau \right),$$

where τ controls the sharpness of the update. A flow-matching actor can then be trained to transport the base distribution toward this advantage-weighted target. This is useful because the target may be multimodal: several separated actions may have high advantage, and a Gaussian actor may fail to represent them without collapsing to an average or selecting only one mode.

FMER is particularly relevant because it combines an ODE-based flow-matching policy, a mirror-descent interpretation of advantage-weighted conditional flow-matching, and an action-space entropy correction for the tanh transformation used in bounded continuous control [30]. The tanh correction is important because many continuous control policies sample an unconstrained variable *u* and map it to a bounded action a=tanh(u). The action-space log density differs from the pre-squash density by a Jacobian term:

(50) $$\log\pi_{a}\left(a|s\right)=\log\pi_{u}\left(u|s\right)-\sum_{i}\log\left(1-\tanh^{2}\left(u_{i}\right)\right),\;a=\tanh\left(u\right).$$

Consequently, the action-space entropy is not the same as the entropy of the pre-squash variable. Ignoring this correction can misrepresent how stochastic the deployed policy actually is in the bounded action space.

ODE-based flow policies therefore connect three themes of this review. First, they address representational limits of Gaussian actors by allowing expressive multimodal action distributions. Second, they preserve a principled route to likelihood and entropy through change in variables and divergence integration. Third, they connect naturally to KL-constrained and mirror-descent policy improvement because an advantage-weighted target distribution can be learned through conditional flow-matching. The remaining challenge is practical rather than conceptual: accurate entropy estimation requires stable ODE integration, reliable divergence estimation, and careful treatment of action squashing, all under the noisy value estimates typical of RL.

The broader lesson is that entropy in generative policy classes is not determined only by an explicit entropy bonus. It depends on the architecture of the sampler, the tractability of likelihood evaluation, the geometry of the latent-to-action map, the use of guidance or advantage weighting, and the numerical estimators used during training. Generative actors therefore make entropy both more powerful and more fragile: they can represent richer uncertainty than simple policies, but controlling that uncertainty requires understanding the full generative mechanism.

## 8. Entropy in Reinforcement Learning for Language Models

Reinforcement learning from human feedback (RLHF) and reinforcement learning with verifiable rewards (RLVR) apply policy optimization to autoregressive language models. A language model defines a policy over token sequences:

(51) $$\pi_{\theta }\left(y|x\right)=\prod_{t=1}^{T}\pi_{\theta }\left(y_{t}|x,y_{<t}\right),$$

where *x* is the prompt, $y=(y_{1},\ldots ,y_{T})$ is the generated response, and $\pi_{\theta }\left(y_{t}|x,y_{<t}\right)$ is a categorical distribution over the vocabulary. In RLHF, the reward is typically learned from preference data; in RLVR, the reward is obtained from a verifier, such as an exact answer checker, unit tests, or a rule-based evaluator. Policy optimization is commonly performed with Proximal Policy Optimization (PPO), Group Relative Policy Optimization (GRPO), Reinforcement Learning with Leave-One-Out baselines (RLOO), REINFORCE-style estimators, or preference-optimization alternatives [34,119,120,121,122,123,124].

The policy is categorical over the vocabulary, so token entropy is easy to compute:

(52) $$H_{t}\left(x,y_{<t}\right)=-\sum_{v\in V}\pi_{\theta }\left(v|x,y_{<t}\right)\log\pi_{\theta }\left(v|x,y_{<t}\right),$$

where V is the vocabulary. A response-level average can be written as

(53) $$\bar{H}\left(y|x\right)=\frac{1}{T}\sum_{t=1}^{T}H_{t}\left(x,y_{<t}\right).$$

However, easy computation does not imply easy interpretation. Token entropy is local: it measures uncertainty over the next token conditioned on the current prefix. Reasoning quality is global: it depends on whether the sequence follows a useful solution path, makes correct intermediate decisions, and reaches a correct final answer. A model can have high entropy over stylistic variants or filler tokens without meaningful reasoning diversity. Conversely, a model can have low entropy at routine tokens while retaining useful uncertainty at pivotal reasoning steps.

Large language models trained for instruction following and reasoning already contain rich priors from pre-training and supervised fine-tuning [125,126,127,128,129,130,131]. RL does not start from a blank policy; it sharpens, suppresses, or redirects an existing distribution. This makes entropy dynamics different from classical continuous control exploration. In a pre-trained LLM, low-probability tokens may include genuinely useful rare reasoning moves, but also errors, irrelevant continuations, or unsafe outputs. High-probability tokens may encode strong linguistic priors, but may also reflect premature convergence to familiar templates. RL can improve reward by increasing probability mass on successful completions, but the same update can reduce response diversity and eliminate alternative solution strategies.

Reasoning-focused models and prompting methods make this issue central. Chain-of-thought prompting, self-consistency, tree search, ReAct, Reflexion, self-refinement, and bootstrapped reasoning methods show that multiple reasoning paths can be valuable even when only one final answer is required [132,133,134,135,136,137,138,139]. The benefit of sampling multiple responses, as in Pass@K evaluation or self-consistency, depends on the model retaining enough response-level diversity to generate different plausible solution paths. If RL collapses the policy too aggressively, Pass@1 may improve while Pass@K, calibration, and robustness degrade. Verifiable reward training on mathematics and coding benchmarks further increases the importance of preserving alternative solution paths because a single prompt may admit multiple valid derivations, programs, or proof strategies [35,36,37,140,141,142,143].

The sequence-level RL objective is usually written in terms of rewards assigned to complete responses:

(54) $$J\left(\theta \right)=E_{y∼\pi_{\theta }\left(\cdot |x\right)}\left[R\left(x,y\right)\right].$$

A KL-regularized version constrains the updated policy relative to a reference model $\pi_{\mathrm{ref}}$:

(55) $$J_{\mathrm{KL}}\left(\theta \right)=E_{y∼\pi_{\theta }\left(\cdot |x\right)}\left[R\left(x,y\right)-\beta D_{\mathrm{KL}}\left(\pi_{\theta }\left(\cdot |x\right)∥\pi_{\mathrm{ref}}\left(\cdot |x\right)\right)\right].$$

In practice, this sequence-level KL is often implemented or estimated through token-level log-probability ratios. The resulting update geometry is important: token probabilities are changed locally, but rewards are often sparse and sequence-level. This mismatch can amplify a few sampled successful tokens and suppress unsampled alternatives, even when those alternatives would also lead to correct solutions.

### 8.1. Entropy Collapse

Recent work, including preprint and peer-reviewed studies, reports that token entropy can drop rapidly during training [31,32,33]. Entropy collapse refers to the concentration of probability mass onto a narrower set of tokens, prefixes, or complete responses. It is not identical to improved confidence. Some entropy reduction is expected and desirable when the model learns to avoid wrong answers. Collapse becomes problematic when probability mass concentrates before the policy has adequately explored the space of valid reasoning strategies, or when the model becomes overconfident on brittle patterns.

The Entropy Mechanism study describes an empirical performance–entropy relation and argues that policy performance may saturate as entropy is exhausted [31]. In this view, entropy is a finite training resource: early RL updates can convert uncertainty into reward improvement, but once the policy distribution becomes too concentrated, additional optimization may produce smaller gains or overfitting. The relevant point is not that entropy should remain high everywhere. Rather, collapse can remove the alternatives that RL needs for continued improvement, especially when rewards are sparse and only sampled responses receive feedback.

Reasoning with Exploration observes that high-entropy regions often correspond to pivotal tokens, reflective actions, and rare reasoning behaviors [32]. This suggests that entropy is not uniformly valuable across a response. Some tokens are mechanically predictable, such as punctuation, common connective phrases, or format markers. Other tokens determine the direction of the reasoning trajectory: choosing a theorem, decomposition, intermediate variable, algorithmic branch, or corrective reflection. Entropy at these pivotal positions may support exploration over solution strategies. Entropy at irrelevant positions may only increase surface variability.

Revisiting Entropy in RL for Large Reasoning Models emphasizes that entropy collapse is associated with response-diversity loss, calibration degradation, clipping thresholds, off-policy updates, and dataset diversity [33]. This connects entropy to several evaluation axes. A model may become more accurate on the training distribution while less calibrated on uncertain prompts. It may produce shorter or more stereotyped reasoning traces. It may preserve token entropy in aggregate while losing diversity over complete responses. These effects show why entropy trajectories should be reported alongside reward, accuracy, length, Pass@K, and calibration metrics.

A useful diagnostic distinction is between token entropy and response diversity. Token entropy is local and can be averaged over positions. Response diversity concerns the distribution over complete outputs:

(56) $$H\left(\pi_{\theta }\left(\cdot |x\right)\right)=-\sum_{y}\pi_{\theta }\left(y|x\right)\log\pi_{\theta }\left(y|x\right),$$

which is generally infeasible to compute exactly for long sequences. Practical proxies include distinct response counts, pairwise semantic diversity, solution-path diversity, Pass@K, self-consistency variance, and the entropy trajectory of sampled tokens. These proxies are not equivalent. A model can have high token entropy but low semantic diversity if variations are superficial. It can also have modest token entropy but high response diversity if uncertainty is concentrated at branching points.

These works collectively move entropy from a background regularizer to a primary diagnostic for post-training dynamics. In classical RL, entropy is often monitored as an auxiliary curve. In RLVR, entropy can indicate whether training is preserving the model’s ability to search over reasoning paths. Entropy collapse is therefore not only an optimization phenomenon; it is also an evaluation and reliability issue.

### 8.2. Token-Level Entropy Versus Useful Reasoning Uncertainty

High entropy over arbitrary tokens is not necessarily useful. The relevant question is whether uncertainty occurs at decision points that affect reasoning trajectories. A token with high entropy may correspond to a meaningful branch in the solution, but it may also reflect uncertainty over wording, formatting, or irrelevant continuations. Similarly, low entropy may indicate a correct confident step, but it may also indicate memorized or overfit reasoning. Entropy must therefore be interpreted together with token role, reward sensitivity, and downstream correctness.

This distinction can be expressed through the policy-gradient update. For a sampled response *y*, a REINFORCE-style update contains terms of the form

(57) $$\nabla_{\theta }J\left(\theta \right)\approx \sum_{t=1}^{T}A_{t}\nabla_{\theta }\log\pi_{\theta }\left(y_{t}|x,y_{<t}\right),$$

where $A_{t}$ is an advantage or return-derived learning signal. If a high-probability token receives positive advantage, the update can further increase its probability and reduce entropy around that prefix. If alternative tokens are not sampled, they receive no direct positive evidence, even if they might also produce correct solutions. Over repeated updates, this can concentrate the policy on sampled successful patterns and suppress unsampled alternatives.

Entropy-based advantage shaping differs from classical entropy regularization because it does not necessarily add an entropy-gradient term to the objective. Instead, detached token entropy can be used to modulate the magnitude of the policy-gradient update [32]. A stylized form is

(58) $${\tilde{A}}_{t}=A_{t}+\lambda \;\mathrm{stopgrad}\left(g\left(H_{t}\right)\right),$$

where *g* may clip, normalize, or otherwise transform token entropy. Because the entropy signal is detached, it changes which sampled tokens receive stronger updates without directly differentiating through the entropy itself. This can focus learning on high-entropy positions that are more likely to represent consequential reasoning choices.

Covariance-based methods similarly focus on tokens whose probability and advantage jointly drive collapse [31]. The relevant mechanism is not merely that a token has high probability or high advantage in isolation, but that probability changes and advantage estimates interact in a way that systematically reduces entropy. Identifying high-covariance tokens allows the algorithm to restrict or reshape updates that would otherwise collapse the distribution too quickly. This approach treats entropy collapse as an update-level phenomenon rather than as a static property of the policy.

Positive-advantage reweighting targets the positive-advantage tokens that amplify sampled high-probability choices and suppress unsampled alternatives [33]. In sparse verifiable reward settings, successful sampled responses can receive strong positive advantage. If these responses are already likely under the model, the update can sharpen existing preferences rather than discover new reasoning strategies. Reweighting positive-advantage contributions can slow this sharpening and preserve alternatives longer. This is conceptually close to support-aware regularization in offline RL: the method does not maximize randomness everywhere, but tries to prevent destructive concentration caused by biased or narrow update signals.

The main lesson is that entropy in LLM RL must be localized and interpreted. Useful entropy is not generic unpredictability; it is uncertainty over meaningful reasoning alternatives. Token-level entropy is valuable when it coincides with branching decisions, rare but correct strategies, reflective corrections, or underexplored solution paths. It is less valuable when it reflects formatting variation, verbosity, or indecision unrelated to the reward. This is why entropy-aware RLVR methods increasingly combine entropy with advantage structure, clipping behavior, covariance diagnostics, response diversity, and calibration rather than relying on a scalar entropy bonus alone.

## 9. Entropy Control Mechanisms for Reasoning LLMs

Entropy control methods for RLVR can be grouped by intervention point. Some methods modify the objective directly by adding entropy or KL terms. Others modify the policy-gradient surrogate through clipping, reweighting, or advantage shaping. A third group intervenes at the diagnostic level by identifying tokens or updates that are responsible for entropy collapse. These mechanisms are related, but they are not interchangeable: direct entropy regularization changes the target objective, clipping changes which likelihood-ratio updates are active, covariance-based methods change which token updates are trusted, and advantage shaping changes the learning signal assigned to sampled tokens.

A useful starting point is the token-level policy-gradient objective. For a prompt *x* and response $y=(y_{1},\ldots ,y_{T})$, a generic RLVR update can be written as

(59) $$L_{\mathrm{PG}}\left(\theta \right)=-E_{y∼\pi_{\theta_{\mathrm{old}}}\left(\cdot |x\right)}\left[\sum_{t=1}^{T}A_{t}\log\pi_{\theta }\left(y_{t}|x,y_{<t}\right)\right],$$

where $A_{t}$ is a token-level or response-level advantage assigned to the sampled token. Entropy collapse occurs when repeated updates concentrate probability mass on a shrinking set of sampled high-reward continuations. The goal of entropy control is therefore not simply to keep $H_{t}$ large everywhere, but to prevent destructive concentration while still allowing the policy to exploit reliable reward signals.

### 9.1. Direct Entropy Regularization and Adaptive Entropy Control

The most direct intervention is to add an entropy bonus to the RL objective:

(60) $$L_{\mathrm{ent}}\left(\theta \right)=L_{\mathrm{PG}}\left(\theta \right)-\alpha E_{x,y}\left[\sum_{t=1}^{T}H\left(\pi_{\theta }\left(\cdot |x,y_{<t}\right)\right)\right],$$

where α>0 controls the strength of entropy regularization. This is the closest analogue of classical maximum-entropy RL. It directly discourages premature determinism by increasing the objective value of broader token distributions. However, direct entropy regularization is blunt. It treats all token positions similarly unless additional weighting is introduced, even though entropy at punctuation, formatting tokens, or boilerplate phrases may be much less important than entropy at reasoning branch points.

Adaptive entropy regularization attempts to reduce this brittleness by changing α during training. One can view this as a target entropy mechanism:

(61) $$\alpha \leftarrow \alpha +\eta_{\alpha }\left({\bar{H}}_{\mathrm{target}}-{\bar{H}}_{\mathrm{batch}}\right),$$

where ${\bar{H}}_{\mathrm{batch}}$ is the observed batch entropy and ${\bar{H}}_{\mathrm{target}}$ is a desired entropy level. If entropy falls below the target, the regularization strength increases; if entropy remains high, the regularization weakens. This mirrors automatic temperature adaptation in continuous control methods such as SAC, but the LLM setting is more delicate because a single target entropy may not capture the difference between useful reasoning uncertainty and irrelevant token-level randomness.

### 9.2. KL Penalties and Reference-Policy Constraints

KL regularization constrains the updated policy relative to a reference model, typically the supervised fine-tuned model or a previous policy checkpoint. A common sequence-level objective is

(62) $$\max_{\theta }\;E_{y∼\pi_{\theta }\left(\cdot |x\right)}\left[R\left(x,y\right)-\beta D_{\mathrm{KL}}\left(\pi_{\theta }\left(\cdot |x\right)∥\pi_{\mathrm{ref}}\left(\cdot |x\right)\right)\right],$$

where β>0 controls the strength of the reference constraint. In tokenized implementations, this penalty is often approximated through token-level log-probability differences:

(63) $$\sum_{t=1}^{T}\left[\log\pi_{\theta }\left(y_{t}|x,y_{<t}\right)-\log\pi_{\mathrm{ref}}\left(y_{t}|x,y_{<t}\right)\right].$$

KL regularization can slow entropy collapse by discouraging large movement away from the reference distribution. It also protects against reward hacking and distributional drift. However, KL is not the same as entropy. A policy can remain close to the reference while still losing diversity in specific regions, and a strong KL penalty can preserve undesirable reference-model habits. Thus, KL control is best interpreted as a distribution shift constraint rather than a direct entropy-preservation mechanism.

The direction of the KL also matters. Reverse-KL-like behavior is mode-seeking and can concentrate on a subset of high-probability responses. Forward-KL-like behavior is more mode-covering and can preserve broader support. RLHF and RLVR implementations often use practical approximations whose entropy effects depend on sampling, advantage estimation, clipping, and reference-model choice. This is why entropy should be monitored directly even when a KL penalty is present.

### 9.3. PPO, GRPO, and Clipping-Based Entropy Control

PPO and GRPO control policy updates through likelihood-ratio clipping. For a sampled token, define

(64) $$r_{t}\left(\theta \right)=\frac{\pi_{\theta }\left(y_{t}|x,y_{<t}\right)}{\pi_{\theta_{\mathrm{old}}}\left(y_{t}|x,y_{<t}\right)}.$$

A token-level clipped surrogate has the form

(65) $$L_{\mathrm{clip}}\left(\theta \right)=-E\left[\min(r_{t}\left(\theta \right)A_{t},\mathrm{clip}\left(r_{t}\left(\theta \right),1-\epsilon ,1+\epsilon \right)A_{t})\right].$$

For positive-advantage tokens, the upper clipping bound limits how much their probability can increase. For negative-advantage tokens, the lower bound limits how much their probability can decrease. These clipping operations affect entropy indirectly. If high-probability positive-advantage tokens repeatedly receive updates, the policy may still become sharper, but clipping slows the rate of concentration. If clipping is too restrictive, useful low-probability reasoning alternatives may not grow enough. If clipping is too loose, entropy collapse can accelerate.

GRPO modifies the advantage estimation structure by using group-relative rewards rather than a learned value baseline, but the entropy control issue remains similar: sampled responses with high relative reward receive positive updates, and sampled responses with low relative reward receive negative updates [34]. In reasoning tasks, this can produce strong selection pressure on the few sampled solution paths that pass the verifier. The policy may improve rapidly, but the update can also suppress unsampled alternatives that were not evaluated. Thus, clipping in PPO and GRPO is a local trust-region mechanism, not a guarantee of response-level diversity.

Clip-Higher-style methods alter clipping asymmetrically to preserve updates on low-probability positive-advantage tokens [7,34,36]. The motivation is that rare but useful tokens may need room to grow. If the same upper clipping threshold is applied uniformly, low-probability tokens with positive advantage may be prevented from receiving sufficiently large corrective updates. Increasing or adapting the upper bound can help preserve exploration over underrepresented reasoning paths. The risk is update aggressiveness: loosening the clipping bound can improve discovery but may also destabilize training or amplify noisy reward signals.

### 9.4. Covariance-Based Control

Covariance-based methods treat entropy collapse as an update-level phenomenon. The central observation is that collapse is driven not only by high token probabilities or high advantages separately, but by their interaction. Tokens whose probability changes are strongly aligned with advantage estimates can dominate the update and rapidly concentrate the distribution. Clip-Cov and KL-Cov therefore restrict high-covariance tokens that are likely to drive entropy collapse [31].

At a high level, these methods examine relationships of the form

(66) $$\mathrm{Cov}(A_{t},\Delta \log\pi_{\theta }\left(y_{t}|x,y_{<t}\right)),$$

or related covariance quantities between advantages, logits, probabilities, and policy-ratio changes. Tokens that contribute disproportionately to entropy reduction can then be clipped, penalized, or otherwise controlled. This differs from a scalar entropy bonus. Rather than asking the model to be more uncertain everywhere, covariance-based control identifies which parts of the update are causing harmful concentration.

The advantage of this approach is specificity. If entropy collapse is caused by a small subset of tokens with strong positive covariance, then global entropy regularization may be inefficient: it increases randomness broadly while failing to target the collapse driver. Covariance control can intervene where the update is most destructive. The limitation is diagnostic reliability. Covariance estimates can be noisy, especially with small sample groups, sparse rewards, long responses, or nonstationary training data. The method therefore depends on stable estimation of token-level update statistics.

### 9.5. Entropy-Based Advantage Shaping

Entropy-based advantage shaping changes the advantage signal rather than adding a direct entropy-gradient term. A stylized form is

(67) $${\tilde{A}}_{t}=A_{t}+\lambda \;\mathrm{stopgrad}\left\{g\left(H_{t}\right)\right\},$$

where $H_{t}=H\left(\pi_{\theta }\left(\cdot |x,y_{<t}\right)\right)$, *g* is a clipping or normalization function, and stopgrad indicates that the entropy term is detached from differentiation. The policy-gradient loss then uses ${\tilde{A}}_{t}$ in place of $A_{t}$:

(68) $$L_{\mathrm{EA}}\left(\theta \right)=-E\left[\sum_{t=1}^{T}{\tilde{A}}_{t}\log\pi_{\theta }\left(y_{t}|x,y_{<t}\right)\right].$$

Because the entropy signal is detached, the method does not directly maximize entropy. Instead, it uses entropy as a token-level indicator of where stronger or weaker learning signals should be applied [32].

This distinction is important. Classical entropy regularization changes the objective by differentiating through $H_{t}$. Entropy-based advantage shaping changes the weighting of sampled token updates. It can prioritize high-entropy positions that are more likely to correspond to uncertain or pivotal reasoning choices. However, it can also misfire if high entropy corresponds to irrelevant uncertainty. For example, if entropy is high because the model is unsure about formatting or verbose filler, increasing the update magnitude there may not improve reasoning. Effective use therefore requires clipping, normalization, or coupling with token-role diagnostics.

### 9.6. Positive-Advantage Reweighting

Positive-advantage reweighting changes the loss weights assigned to positive-advantage tokens [33]. The motivation is that entropy collapse can be driven by repeatedly increasing the probability of sampled high-reward tokens. In sparse verifiable reward settings, a response that passes the verifier may assign positive advantage to many tokens in the sampled trajectory. If those tokens are already likely, the update sharpens the model’s existing preference and suppresses alternatives that were not sampled. Over time, this can reduce response diversity even when the model continues to improve reward.

A generic reweighted objective can be written as

(69) $$L_{\mathrm{PAR}}\left(\theta \right)=-E\left[\sum_{t=1}^{T}w_{t}A_{t}\log\pi_{\theta }\left(y_{t}|x,y_{<t}\right)\right],$$

where $w_{t}$ is chosen to reduce the collapse-inducing effect of positive-advantage updates. For example, $w_{t}$ may be smaller for high-probability positive-advantage tokens or may depend on an entropy, ratio, or confidence statistic. The goal is not to prevent learning from successful responses, but to avoid over-amplifying the tokens that most rapidly concentrate probability mass.

This mechanism is conceptually related to support-aware offline RL. In offline RL, the danger is assigning high value to unsupported actions. In RLVR, the danger is over-reinforcing sampled successful tokens while suppressing unsampled but potentially valid alternatives. Both settings require policy improvement under incomplete evidence. Positive-advantage reweighting addresses this by slowing the concentration induced by positive sampled evidence.

### 9.7. Practical Comparison of Intervention Points

The main entropy control mechanisms differ in what they modify. Entropy regularization modifies the objective. KL penalties modify distance from a reference policy. PPO and GRPO clipping modify likelihood-ratio updates. Clip-Higher changes clipping asymmetry to help low-probability positive-advantage tokens. Covariance-based methods identify update components that drive entropy collapse. Entropy-based advantage shaping modifies token-level learning signals. Positive-advantage reweighting modifies the weights of positive-advantage terms.

These mechanisms can be complementary. A KL penalty can prevent large reference drift, while clipping controls local update ratios. Entropy diagnostics can identify collapse even when KL remains bounded. Advantage shaping can emphasize high-entropy reasoning positions, while positive-advantage reweighting can prevent over-sharpening on sampled successes. However, stacking mechanisms without diagnostics can obscure the cause of improvement or failure. An entropy-aware RLVR system should therefore report not only reward and accuracy, but also token entropy trajectories, response diversity, KL to reference, clipping fractions, advantage distributions, Pass@K, calibration, and length statistics.

The central design principle is that entropy should be controlled at the point where collapse is produced. If collapse is caused by global over-optimization, direct entropy or KL regularization may be appropriate. If collapse is caused by clipping asymmetry, clipping thresholds should be adjusted. If a small set of tokens drives the entropy drop, covariance-based control is more targeted. If useful uncertainty is localized at reasoning branch points, entropy-based advantage shaping may be preferable. If positive-advantage updates over-amplify sampled successes, reweighting those terms can preserve alternatives. This intervention point view prevents treating entropy as a single scalar knob and aligns entropy control with the actual mechanics of LLM policy optimization.

## 10. Evaluation, Calibration, and Diagnostics

Entropy-aware algorithms should not be evaluated by return alone. A final reward curve can show whether an algorithm solved a benchmark, but it usually does not explain how entropy affected learning. Entropy may improve exploration, stabilize policy improvement, preserve behavioral diversity, or prevent premature collapse. It may also inject unproductive randomness, violate offline support, obscure critic error, or increase response variability without improving reasoning. Evaluation must therefore separate task performance from the mechanism by which entropy changes the learned policy.

In continuous control, standard benchmarks such as MuJoCo, OpenAI Gym, and large-scale distributed RL systems provide useful return curves but often hide whether entropy improves coverage or merely injects noise [144,145,146,147,148,149,150,151,152,153]. Two algorithms can reach similar returns while having very different entropy trajectories. One may maintain broad exploration and gradually specialize; another may collapse early and succeed only because the benchmark admits a narrow deterministic strategy. Conversely, an algorithm with higher entropy may show slower reward improvement even though it discovers more diverse behaviors. Reporting only return therefore risks confusing exploration quality with final exploitation.

A basic diagnostic suite for continuous control entropy should include policy entropy, temperature dynamics, state–action coverage, critic uncertainty, and seed variance. For stochastic actors, one can report

(70) $${\bar{H}}_{\pi }=E_{s∼d^{\pi }}\left[H\left(\pi \left(\cdot |s\right)\right)\right],$$

together with the learned temperature α when automatic entropy tuning is used. Coverage can be measured through state visitation statistics, occupancy-density estimates, trajectory clustering, or task-specific behavioral descriptors. Critic uncertainty can be assessed through ensembles, disagreement, temporal-difference error, or calibration of value estimates. Seed variance is essential because entropy bonuses often interact strongly with initialization, replay buffer composition, and early exploration. An entropy method that improves the best seed while worsening median performance should not be interpreted as reliably improving exploration.

Offline RL requires additional diagnostics because high entropy can be harmful outside the dataset support. Evaluation should report not only return, but also behavior-policy deviation, out-of-distribution action rates, conservative value estimates, and sensitivity to dataset quality. A support-aware diagnostic can measure a divergence or distance between the learned policy and the empirical behavior distribution:

(71) $$\Delta_{\mathrm{support}}=E_{s∼D}\left[D\left(\pi \left(\cdot |s\right),\pi_{\beta }\left(\cdot |s\right)\right)\right],$$

where *D* may be a KL divergence, maximum mean discrepancy, learned discriminator score, or likelihood under a behavior model. This quantity should be interpreted together with return. A policy that improves return while moving moderately within support may be genuinely better; a policy that improves estimated value while assigning high probability to unsupported actions may be exploiting critic error.

For generative policies, evaluation should distinguish sampling diversity from tractable entropy. Diffusion, flow, and sequence model policies may generate diverse actions or trajectories, but diversity alone does not imply calibrated likelihood or useful exploration. Useful diagnostics include sample diversity, likelihood or surrogate likelihood, entropy estimates when available, guidance strength, number of denoising or ODE steps, action-squashing corrections, and sensitivity to sampling temperature. For ODE-based flow policies, divergence-estimator variance and solver tolerance should also be reported because they affect likelihood and entropy estimates. A generative actor should therefore be evaluated as both a policy and a sampler.

For LLMs, evaluation should include Pass@1, Pass@K, response diversity, calibration, length distributions, repetition rates, and out-of-domain generalization. Pass@1 measures single-sample performance, whereas Pass@K measures whether the model retains enough diversity to produce a correct answer among multiple attempts. The gap between Pass@1 and Pass@K is particularly informative: a large gap may indicate useful remaining exploration, while a shrinking gap may indicate either improved single-sample reliability or collapse of alternative reasoning paths. Response diversity should be measured semantically, not only lexically, because superficial wording changes do not necessarily represent different solution strategies.

Calibration is central because entropy collapse can produce overconfident wrong answers. For classification-like or verifiable tasks, calibration can be measured through expected calibration error (ECE), confidence–accuracy curves, selective prediction, or the separation between log probabilities assigned to correct and incorrect outputs. A model whose token probabilities become sharper during RL may appear more decisive, but decisiveness is not the same as reliability. In reasoning tasks, calibration should be evaluated both at the final-answer level and, when possible, at intermediate reasoning steps. This is especially important for mathematical and coding tasks where a response can contain plausible reasoning but fail because of a local error.

Length and repetition diagnostics are also necessary. Entropy control methods can change the distribution of response lengths, the frequency of reflective phrases, and the tendency to repeat or over-explain. A method that improves Pass@K by generating longer responses may not represent a genuine improvement in reasoning efficiency. Similarly, entropy regularization may increase surface variation without increasing solution diversity. Reporting length-normalized rewards, repetition rates, and token-position entropy curves can help separate meaningful exploration from verbosity.

Out-of-domain evaluation is needed because entropy collapse may be hidden on the training distribution. A policy optimized on a narrow set of verifiable tasks may become highly confident on familiar prompt patterns while losing robustness on shifted domains. Evaluation should therefore include held-out problem types, adversarially perturbed prompts, different reasoning formats, and tasks with changed surface structure. For LLMs, this includes measuring whether entropy control methods preserve performance on prompts that require alternative algorithms, uncommon reasoning strategies, or longer-horizon planning.

The connection to broader language model evaluation and scaling is important: large models, instruction tuning, constitutional training, debate, scalable oversight, and alignment data shape the initial entropy landscape before RL begins [154,155,156,157,158,159,160,161,162,163,164]. A post-training algorithm does not create entropy from nothing; it reshapes the uncertainty inherited from pre-training and supervised fine-tuning. Scaling-law work provides a useful analogy, but entropy-collapse studies suggest that post-training compute can be bottlenecked by remaining exploratory capacity [165,166,167]. If RL consumes the useful diversity of the policy too early, more optimization may produce diminishing returns even when additional verifier feedback is available.

A practical evaluation protocol should therefore report four classes of quantities. First, performance metrics such as return, accuracy, Pass@1, Pass@K, and sample efficiency. Second, entropy and diversity metrics such as policy entropy, token entropy, response diversity, state coverage, trajectory diversity, and mode counts. Third, reliability metrics such as calibration, uncertainty-quality correlation, critic uncertainty, and out-of-distribution (OOD) generalization. Fourth, mechanism diagnostics such as clipping fraction, KL to reference, temperature trajectory, advantage distribution, covariance statistics, guidance strength, and support deviation. These quantities make it possible to determine whether entropy is improving learning or merely changing the appearance of the policy distribution.

Table 5 reformulates these metrics as diagnostic questions, emphasizing what can go wrong if each axis is omitted.

The main evaluation principle is that entropy should be judged by its effect on controlled uncertainty. In online RL, this means uncertainty that improves exploration and value learning. In offline RL, it means uncertainty that remains inside data support. In generative policies, it means diversity that is represented by a well-understood sampler and, when needed, a tractable density. In LLM RL, it means uncertainty over meaningful reasoning alternatives rather than arbitrary token variation. A complete diagnostic suite should therefore connect entropy measurements to reward, diversity, calibration, support, and robustness rather than treating entropy as an isolated scalar.

## 11. Comparative Synthesis and Method-Selection Guidance

This section consolidates the preceding survey into a literature-grounded comparison of entropy mechanisms, diagnostics, and selection criteria, with the goal to help readers decide which entropy control strategy is appropriate for a given learning regime.

Table 6 summarizes how representative policy optimization families introduce entropy or entropy-like constraints.

### 11.1. Comparison Axes

Entropy control methods should be compared along four axes rather than by final return alone. First, the *entropy object* must be identified: action entropy, trajectory entropy, occupancy entropy, latent entropy, path entropy, token entropy, or response-level diversity. Second, the *reference distribution* must be specified: the previous policy, a behavior policy, a demonstration distribution, a pre-trained language model, or no explicit reference. Third, the *failure mode* should be stated: premature determinism, unsupported extrapolation, poor calibration, mode collapse, meaningless stochasticity, or unstable policy updates. Fourth, the *diagnostics* should match the mechanism: state coverage for exploration, support violation for offline RL, likelihood or entropy-estimation error for generative policies, and Pass@K/diversity/calibration for reasoning LLMs.

Table 7 summarizes these distinctions. The table is intended as a qualitative synthesis of the reviewed literature, not as a new empirical ranking.

### 11.2. Practical Selection Rules

For *online RL*, entropy regularization is most useful when the dominant problem is premature policy collapse or insufficient exploration. Adaptive-temperature maximum-entropy methods are generally preferable to a fixed entropy coefficient because reward scales and action dimensions vary across tasks [3,168]. However, action entropy should not be treated as a sufficient exploration metric: environments that require long-horizon commitment, sparse rewards, or temporally extended exploration often require intrinsic motivation, skill discovery, or state-coverage objectives in addition to local action stochasticity [103,105].

For *offline RL*, entropy should be used conservatively. The relevant question is not whether the learned policy is stochastic, but whether its stochasticity remains inside reliable dataset support. Methods such as CQL, IQL, BCQ, BEAR, and related support-aware approaches differ in how they constrain policy improvement, value extrapolation, or behavior-policy deviation [16,17,18,19]. When the dataset has narrow support, explicit conservatism or behavior regularization is usually safer than maximizing entropy directly.

For *generative policies*, the selection criterion is the trade-off between expressiveness and tractability. Gaussian policies are attractive when closed-form entropy and likelihood ratios are needed. Diffusion and flow-based policies are useful when action distributions are multimodal or structured, but they complicate entropy estimation, KL control, and likelihood-ratio policy gradients unless the model class provides tractable density or reliable estimators [23,27,29,85]. Thus, expressive generative actors should be selected when multimodality is essential, not merely because they produce diverse samples.

For *LLM reasoning*, entropy control should be tied to the evaluation objective. If the goal is single-sample accuracy, excessive entropy can hurt by preserving low-probability or low-quality reasoning paths. If the goal is Pass@K, robustness, or exploration over solution strategies, preserving response-level diversity can be beneficial. Recent RLVR methods therefore combine KL control, clipping, group-relative normalization, dynamic sampling, entropy diagnostics, or advantage reshaping rather than simply maximizing token entropy [33,34,36,122]. The important diagnostic is not token entropy alone, but whether uncertainty is concentrated at reasoning-relevant branching points.

### 11.3. Recommended Reporting Checklist

To make entropy control comparisons reproducible and interpretable, future empirical work should report:

1. The entropy object being measured and whether it is exact, estimated, or only proxied;
2. The reference distribution or support constraint used by the method;
3. The coefficient, temperature, KL budget, clipping range, or dual-update rule controlling entropy;
4. At least one task performance metric and one entropy/diversity/support diagnostic;
5. Seed variability and sensitivity to the entropy control hyperparameter;
6. For LLMs, whether entropy is measured at the token, sequence, semantic response, or Pass@K level.

This checklist addresses the need for better comparison without adding unsupported numerical results. It also makes explicit that method selection depends on the regime-specific role of entropy: exploration in online RL, support preservation in offline RL, tractable likelihood control in generative policies, and useful reasoning diversity in LLM RL.

## 12. Open Problems and Future Directions

The preceding sections show that entropy has become a unifying but overloaded concept in deep RL. It appears as an exploration bonus, an inference principle, a trust-region mechanism, a support constraint, a diversity objective, a generative-model property, and a collapse diagnostic. This breadth is useful, but it also creates unresolved questions. Future work must move beyond asking whether entropy is “good” or “bad” and instead specify which entropy object is being controlled, what mechanism it induces, and what failure mode it prevents or creates.

### 12.1. Limitations of This Review

This review focuses on entropy regularization in modern deep reinforcement learning, especially policy optimization, maximum-entropy objectives, offline RL, imitation learning, generative policies, and LLM post-training. Related areas such as tabular and linear RL, continuous-time control, Bayesian RL, multi-agent RL, and exhaustive catalogs of value-based variants are therefore outside its main scope.

The discussion of generative policies and LLM post-training is similarly selective: we emphasize methods most directly connected to the entropy-regularized RL perspective developed in the paper. Finally, several recent works in LLM reasoning and RLVR are preprints or technical reports, so their conclusions are treated as preliminary where appropriate.

### 12.2. Useful Exploration Versus Noise

A central open problem is to distinguish useful uncertainty from meaningless randomness. In robotics, entropy over motor commands may not imply diverse state visitation. A stochastic policy can repeatedly perturb actions around the same local behavior while failing to reach new states or discover new skills. Conversely, a temporally coherent low-entropy controller may produce broad exploration if it commits to distinct strategies over long horizons. Thus, action entropy is only a local proxy for exploration quality.

In LLMs, the same issue appears as the difference between token entropy and reasoning diversity. High entropy over syntactic filler, formatting choices, or interchangeable phrases may not improve reasoning. Useful entropy is concentrated at decision points that affect the solution path: selecting a decomposition, choosing an algorithm, introducing an intermediate variable, deciding whether to backtrack, or exploring an alternative proof. Future objectives should therefore weight entropy by controllability, novelty, causal influence on reward, or reasoning role rather than treating all uncertainty equally [32,105,169].

One promising direction is role-conditioned entropy. Instead of maximizing H(π(·|s)) or token entropy uniformly, the algorithm could learn a weighting function

(72) $$J_{\mathrm{role}}\left(\pi \right)=E_{\rho_{\pi }}\left[\sum_{t}r_{t}+\alpha w_{t}H\left(\pi \left(\cdot |s_{t}\right)\right)\right],$$

where $w_{t}$ measures whether uncertainty at time *t* is likely to affect future reward, coverage, controllability, or reasoning correctness. In continuous control, $w_{t}$ could depend on novelty, model uncertainty, or influence on future state visitation. In LLM RL, it could depend on token role, verifier sensitivity, branching importance, or whether a token lies near a reasoning bottleneck. The open challenge is to estimate such weights without introducing new reward-hacking channels.

### 12.3. Entropy Estimation for Expressive Policies

Diffusion policies, energy-based policies, and non-invertible generative actors need entropy estimators that are accurate enough for policy optimization. For simple Gaussian and categorical policies, entropy is tractable. For expressive generative policies, the sampler may be easy to run while the likelihood and entropy are difficult to evaluate. This creates a gap between sample diversity and policy entropy. A policy may generate diverse samples without providing a reliable value of logπ(a|s), making entropy bonuses, KL constraints, likelihood-ratio gradients, and trust-region methods harder to apply.

ODE-based flows and flow-matching provide one route because likelihood and entropy can be connected to the divergence of the learned velocity field [26,27,29,30]. However, divergence estimation, action squashing, numerical solver error, and high-dimensional variance remain difficult. In bounded continuous control, pre-squash entropy can differ substantially from action-space entropy. In high-dimensional action spaces, trace estimators can have high variance. In long-horizon sequence generation, even token-level likelihoods are tractable but sequence-level entropy is generally infeasible to compute exactly.

Future work should develop entropy estimators that are simultaneously accurate, low-variance, differentiable, and compatible with policy optimization. For flow and CNF policies, this may require better divergence estimators, solver-aware regularization, or entropy bounds that remain valid under numerical integration. For diffusion policies, it may require tractable entropy surrogates tied to denoising likelihoods, path-space KLs, or score-matching quantities. For LLMs, it may require better response-level diversity estimators that go beyond average token entropy. The core open problem is to make entropy estimation match the entropy object that actually matters for the task.

### 12.4. Entropy Control Without Reward Hacking

Entropy can be gamed. In language models, rewarding reasoning-like tokens, long chains of thought, or high token entropy can produce verbosity rather than better reasoning. A model may learn to preserve uncertainty in unimportant parts of the response while becoming overconfident at the final answer. In control, diversity objectives can discover irrelevant behaviors that maximize novelty without improving task competence. In offline RL, a high-entropy policy can exploit critic error by assigning probability to unsupported actions.

Robust entropy control should therefore be coupled with verifiable outcomes, calibration, and support constraints [32,33,122,123]. Entropy should not be rewarded in isolation. Instead, it should be evaluated conditionally: does it improve reward, preserve useful alternatives, maintain calibration, or increase robustness under distribution shift? This suggests constrained objectives of the form

(73) $$\max_{\pi }\;J\left(\pi \right)\;s.t.\;H_{\mathrm{useful}}\left(\pi \right)\geq h,\;\Delta_{\mathrm{support}}\left(\pi ,\pi_{\beta }\right)\leq \epsilon ,\;\mathrm{Cal}\left(\pi \right)\leq c,$$

where $H_{\mathrm{useful}}$ is a task-relevant entropy or diversity measure, $\Delta_{\mathrm{support}}$ measures deviation from reliable data support or a reference policy, and Cal measures calibration error. The difficulty is that each constraint is domain-dependent and may be expensive to estimate.

Another open direction is adversarial evaluation of entropy control methods. If an algorithm claims to preserve reasoning diversity, it should be tested on prompts where multiple solution strategies are possible, not only on standard accuracy benchmarks. If an algorithm claims to improve exploration, it should be tested on environments where random action noise is insufficient. If it claims to preserve support, it should report out-of-distribution action rates and critic-error sensitivity. Without such tests, entropy control can appear beneficial while merely shifting failure modes.

### 12.5. Unified Theory Across Continuous and Token Spaces

The common mathematical structure is regularized policy improvement under constrained distribution shift. A unified theory should compare differential action entropy, categorical token entropy, trajectory entropy, occupancy entropy, path entropy, and mutual information within one framework. Such a theory would explain why entropy supports exploration in online control, must be constrained in offline RL, becomes a tractability issue in generative policies, and becomes a collapse diagnostic in RLVR [9,10,52,57,170,171].

One route is to formulate entropy control as choosing three objects: a distribution *q* to regularize, a reference distribution *p* to remain close to, and a divergence or entropy functional Ω(q,p). Classical MaxEnt RL often chooses q=π(·|s) and Ω=−H(π). Trust-region methods choose $q=\pi_{k+1}$, $p=\pi_{k}$, and $\Omega =D_{\mathrm{KL}}\left(\pi_{k+1}∥\pi_{k}\right)$. Offline RL chooses $p=\pi_{\beta }$ or the dataset occupancy. LLM RL chooses $p=\pi_{\mathrm{ref}}$ or $\pi_{\mathrm{old}}$ and applies the penalty tokenwise or sequencewise. Generative policy methods add another layer because *q* may be defined implicitly by a sampler.

A unified theory should also account for granularity. Entropy at the action level, trajectory level, occupancy level, latent level, and token level can move in different directions during training. For example, token entropy may decrease while response-level diversity remains high if uncertainty is concentrated at a small number of branching positions. Action entropy may remain high while state coverage remains poor. Latent entropy may be high while action entropy is reduced by a squashing function. These mismatches suggest that future theory should treat entropy as a multilevel quantity rather than a scalar diagnostic.

### 12.6. Benchmarking Entropy Mechanisms Rather than Only Algorithms

Another open problem is benchmark design. Current benchmarks often evaluate final performance but do not isolate the mechanism by which entropy helps or hurts. A useful entropy benchmark should distinguish at least four cases: environments where local action entropy is sufficient, environments requiring temporally extended exploration, offline datasets where high entropy violates support, and reasoning tasks where useful diversity occurs at sparse branching points. Without such benchmarks, algorithms may appear broadly entropy-aware while solving only one narrow version of the problem.

For continuous control, benchmarks should report state coverage, trajectory diversity, action entropy, and robustness across seeds. For offline RL, they should vary dataset support, behavior-policy diversity, and the amount of extrapolation required. For generative policies, they should compare sample diversity, likelihood tractability, entropy-estimation accuracy, and control performance. For LLM RL, they should separate Pass@1, Pass@K, semantic response diversity, calibration, length effects, and OOD reasoning. The goal is not to create more metrics for their own sake, but to make it possible to identify which entropy mechanism is responsible for an observed gain.

### 12.7. Entropy Under Distribution Shift and Deployment Constraints

Entropy control methods are usually developed under training-time assumptions, but deployment changes the meaning of uncertainty. In robotics, a stochastic exploratory policy may be unsafe when deployed on hardware. In offline RL, support constraints derived from a dataset may not match the deployment environment. In LLMs, sampling temperature, decoding rules, safety filters, and user prompts all reshape the effective policy distribution. Entropy measured during training may therefore differ from entropy experienced at deployment.

Future work should study how entropy transfers across training, validation, and deployment distributions. A policy that preserves entropy during RLVR training may still collapse under greedy decoding. A diffusion policy with diverse samples may become narrow under strong value guidance. A continuous control policy with high action entropy may be clipped or filtered by safety controllers. Evaluation should therefore report entropy under the actual deployment sampler, not only under the training objective.

### 12.8. Entropy in Broader Machine Learning

Entropy regularization is not unique to RL; it appears throughout machine learning. Understanding these connections can inform RL entropy control design.  

Variational inference.

The ELBO objective in variational autoencoders contains an entropy term:

(74) $$\mathrm{ELBO}=E_{q(z|x)}\left[\log p\left(x|z\right)\right]+H\left(q\left(z|x\right)\right).$$

The entropy term encourages the variational posterior to be broad, similar to exploration in RL. Connections between variational inference and RL have been explored in control-as-inference formulations [52].  

Information bottleneck.

The information bottleneck method uses mutual information constraints:

(75) $$\max_{p(z|x)}I\left(X;Y\right)-\beta I\left(X;Z\right).$$

The entropy of the representation *Z* is implicitly controlled by the mutual information term. This relates to skill discovery where entropy of skills is encouraged [171].  

Generative models.

GANs and VAEs use entropy-like objectives. Diffusion models use variational bounds that include entropy terms. Understanding entropy in generative modeling informs generative policy design.  

Active learning.

Entropy-based acquisition functions select samples with high uncertainty:

(76) a(x)=H(p(y|x)).

This relates to exploration in RL where entropy encourages visiting uncertain states.  

Bayesian optimization.

Entropy search methods maximize information gain, connecting entropy to experimental design and efficient exploration.  

Multi-agent systems.

Entropy appears in MARL for exploration and cooperation. Social influence and entropy regularization can promote beneficial interactions.  

Distributionally robust optimization.

Entropy-regularized objectives connect to robust optimization through divergences, providing theoretical foundations for entropy control.

These connections suggest that entropy control methods developed in one subfield may transfer to others, and that a unified understanding of entropy across ML could lead to better RL entropy control design.

## 13. Conclusions

Entropy regularization is not a single technique but a family of mechanisms for controlling uncertainty, distribution shift, and diversity. Across the literature reviewed in this paper, entropy appears at different levels: actions, trajectories, occupancies, latent variables, denoising paths, ODE flows, tokens, and complete responses. These objects are mathematically related, but they support different algorithmic goals. Treating them as interchangeable can obscure whether entropy is encouraging useful exploration, preserving support, resolving ambiguity, improving calibration, maintaining reasoning diversity, or merely injecting noise.

Classical maximum-entropy RL uses entropy to smooth Bellman backups, prevent premature deterministic policy improvement, and encourage exploration. Trust-region and mirror-descent methods reinterpret entropy through KL-regularized distributional change, showing that policy improvement is not only about increasing reward but also about controlling how far the policy moves. IRL and imitation learning use maximum-entropy to resolve ambiguity among expert-consistent behaviors, shifting the meaning of entropy from exploration to probabilistic explanation. Offline RL reverses the classical lesson: entropy is useful only when it remains inside dataset support, because unsupported stochasticity can exploit critic error and degrade deployment performance.

Intrinsic motivation and skill discovery methods broaden the concept further by replacing local action randomness with coverage, novelty, mutual information, controllability, and behavioral diversity. These methods show that high policy entropy is not equivalent to useful exploration. Temporally extended skills may require low local entropy but high trajectory diversity, while random actions may have high entropy and low value. Generative policy classes expose another dimension: entropy becomes a question of tractability. Diffusion policies, normalizing flows, continuous normalizing flows, and flow-matching policies can represent multimodal action distributions, but they differ sharply in whether likelihoods, KL terms, and entropy corrections are available for policy optimization.

Reinforcement learning for language models makes entropy a central post-training diagnostic. In RLHF and RLVR, token entropy is easy to compute but hard to interpret. Entropy collapse can improve short-term reward while reducing response diversity, calibration, Pass@K, and the ability to explore alternative reasoning paths. Recent entropy control mechanisms therefore intervene at different points in the optimization pipeline: direct entropy regularization, KL penalties, PPO/GRPO clipping, asymmetric clipping, covariance-based update control, entropy-based advantage shaping, and positive-advantage reweighting. These methods reinforce the main theme of the review: entropy should be controlled where collapse or harmful concentration is actually produced.

The practical lesson is not to maximize entropy blindly. Entropy must be tied to the correct object, the correct mechanism, and the correct failure mode. In online control, the relevant question is whether entropy improves exploration and value learning. In offline RL, it is whether entropy respects data support. In imitation learning, it is whether entropy represents expert ambiguity. In generative policies, it is whether entropy can be estimated or controlled through the sampler. In LLM RL, it is whether entropy preserves meaningful reasoning alternatives rather than superficial token variation. A useful entropy-aware algorithm should therefore report reward together with entropy trajectories, diversity, calibration, support deviation, clipping behavior, and robustness across seeds or prompts.

Future progress will depend on more localized and mechanistic entropy objectives. Promising directions include role-weighted entropy for reasoning tokens, support-aware entropy for offline policies, tractable entropy estimators for expressive generative actors, and unified theories connecting differential action entropy, categorical token entropy, occupancy entropy, trajectory entropy, and mutual information. The unifying view is regularized policy improvement under constrained distribution shift. Entropy is valuable when it shapes the policy distribution toward useful uncertainty; it is harmful when it hides uncertainty in irrelevant randomness or pushes the policy outside reliable evidence. This distinction should guide the design, diagnosis, and evaluation of entropy-centered deep reinforcement learning systems.   

## Figures and Tables

**Table 1:** Taxonomy of entropy roles across research areas.

Area	Entropy Object	Primary Role	Main Risk
Classical online RL	Action distribution	Exploration and smooth policy improvement	Randomness can slow exploitation
Policy gradients and trust regions	Update-induced policy change	Prevent destructive distribution shift	KL/clipping can induce unintended entropy dynamics
MaxEnt IRL and imitation	Trajectory or occupancy distribution	Resolve expert ambiguity	May imitate broad but irrelevant behavior
Offline RL	Policy relative to dataset support	Avoid out-of-distribution actions	Excess entropy violates support constraints
Intrinsic motivation	State, skill, or behavior diversity	Discover reusable behaviors	Diversity can decouple from task reward
Generative policies	Latent and sampled action distributions	Represent multimodal actions	Entropy may be intractable or biased
LLM RLVR	Token and response distributions	Maintain reasoning exploration	Entropy collapse or unhelpful randomness

**Table 2:** Entropy objects, mechanisms, and representative uses.

Entropy Object	Mathematical Level	Typical Mechanism	Representative Setting
Action entropy	H(π(·|s))	Encourages stochastic exploration and smooth policy improvement	SAC, soft Q-learning, PPO bonuses
Trajectory entropy	H(τ) or causal entropy	Resolves ambiguity among behavior sequences	MaxEnt IRL, control-as-inference
Occupancy entropy	Entropy of $d^{\pi }\left(s,a\right)$	Encourages coverage of state–action space	exploration, imitation, skill discovery
Relative-entropy	$D_{\mathrm{KL}}\left(\pi ∥\pi_{\mathrm{old}}\right)$ or $D_{\mathrm{KL}}\left(\pi ∥\pi_{\beta }\right)$	Constrains policy movement or dataset deviation	TRPO, PPO, MPO, offline RL
Latent-policy entropy	Entropy of latent transport distribution	Supports expressive multimodal action sampling	flows, continuous normalizing flows, flow-matching
Denoising-path entropy	Entropy induced by iterative stochastic sampling	Represents complex action distributions but is often hard to evaluate	diffusion policies
Token entropy	$H(\pi \left(\cdot |x,y_{<t}\right))$	Measures uncertainty over next-token choices	RLHF, RLVR, GRPO, reasoning LLMs
Response diversity	Entropy or diversity over complete generations	Measures exploration of alternative solutions	Pass@K, self-consistency, reasoning search

**Table 3:** Comparison of entropy and related quantities.

Quantity	Definition	Key Property	Use in RL
Shannon entropy	$-\sum p_{i}\log p_{i}$	Maximal at uniform distribution	Standard entropy bonus
Rényi entropy	$\frac{1}{1-\alpha }\log\sum p_{i}^{\alpha }$	Focuses on high/low probability events	Rare-action emphasis
Tsallis entropy	$\frac{1}{q-1}\left(1-\sum p_{i}^{q}\right)$	Non-additive	Heavy-tailed distributions
Cross-entropy	$-\sum p_{i}\log q_{i}$	Measures surprise under *q*	Supervised learning
KL divergence	$\sum p_{i}\log\left(p_{i}/q_{i}\right)$	Asymmetric distance	Trust-region constraint

**Table 4:** Toy MDP illustrating that larger local action entropy need not imply useful exploration. Values are computed by exact trajectory enumeration.

Policy	Return	Local Action Entropy	State Coverage	Useful Branch	Success
Reward only	0.917	0.263	2.950	0.950	0.902
Reward + action entropy	0.340	0.910	2.200	0.200	0.100
Reward + coverage bonus	0.690	0.745	2.650	0.650	0.585

**Table 5:** Evaluation questions for entropy-aware RL.

Question	Metric Family	Failure Mode if Omitted
Does entropy improve reward?	Return, accuracy, sample efficiency	Exploration cost hidden by final score
Does entropy preserve diversity?	State coverage, response diversity, Pass@K	Deterministic collapse masked by Pass@1
Does entropy improve reliability?	Calibration, log probability of correct/incorrect outputs	Overconfidence mistaken for competence
Does entropy respect support?	OOD action rate, behavior KL, conservative Q estimates	High entropy exploits critic error
Does entropy target useful uncertainty?	Token-role analysis, pivotal-token entropy, ablations	Randomness confused with reasoning exploration
Does entropy remain stable across runs?	Seed variance, confidence intervals, entropy trajectories	Single-seed improvement mistaken for robustness
Does entropy interact with the sampler?	Guidance strength, decoding temperature, solver tolerance, denoising steps	Sampling diversity mistaken for policy entropy

**Table 6:** How entropy enters representative policy optimization families.

Family	Mechanism	Entropy Effect	Representative Works
Entropy bonus	Add αH to reward/objective	Directly encourages stochasticity	Policy-gradient bonuses, SAC
KL trust-region	Penalize or constrain policy shift	Indirectly limits collapse and instability	Natural gradient, TRPO, PPO
Mirror-descent	Project advantage-weighted distribution	Balances exploitation with proximity	MPO, AWR, AWAC
Offline behavior regularization	Constrain distance to data policy	Prevents high-entropy OOD actions	BCQ, BEAR, BRAC, CQL, IQL
Token-level RLVR control	Modify clipping, covariance, or advantages	Regulates entropy collapse in LLMs	Dynamic Sampling Policy Optimization (DAPO), Clip-Cov, entropy-advantage shaping, positive-advantage reweighting (PAR)

**Table 7:** Qualitative method-selection guide for entropy control mechanisms.

Regime	Recommended Default	Entropy/Control Object	Main Risk	Useful Diagnostics
Online continuous control	Maximum-entropy actor–critic with adaptive temperature	Action entropy relative to reward scale	Entropy may become action noise rather than state-space exploration	Return, state coverage, action entropy, seed sensitivity
Offline RL	Support-aware objectives or conservative value learning	Policy uncertainty constrained by dataset support	High-entropy actions outside the behavior distribution can exploit critic error	OOD action rate, behavior-policy distance, conservative value gap
Imitation and IRL	Maximum-entropy or occupancy-matching formulations	Trajectory or occupancy entropy	Entropy may explain demonstrations but not guarantee robust deployment	Demonstration likelihood, occupancy mismatch, held-out task transfer
Skill discovery and diversity	Mutual information or diversity objectives	Skill, trajectory, or state-coverage diversity	Diversity can become behaviorally irrelevant	Skill discriminability, coverage, controllability, downstream transfer
Generative policies	Diffusion/flow policies when multimodality matters; Gaussian actors when tractability matters	Latent, path, or induced action entropy	Sample diversity may not imply tractable policy entropy or stable likelihood ratios	Sample diversity, likelihood tractability, entropy-estimation error, control performance
LLM reasoning	KL-controlled policy optimization with entropy diagnostics; explicit diversity shaping only when Pass@K or exploration is important	Token entropy and response-level diversity relative to a reference model	Higher entropy can preserve low-quality or stylistic variation rather than useful reasoning alternatives	Pass@1, Pass@K, semantic diversity, calibration, length-normalized entropy

## Data Availability

No new data were created or analyzed in this study.

## Appendix A. Code for Toy MDP Example

The following Python v3.13.13 code was used to generate the results in Table 4.

import numpy as~np

H=4
eps=1e-12
terminal={3,4}

policies={
"Reward only":{
0:np.array([0.05,0.95]),
1:np.array([1/3,1/3,1/3]),
2:np.array([0.95,0.05])
},
"Reward + action entropy":{
0:np.array([0.80,0.20]),
1:np.array([1/3,1/3,1/3]),
2:np.array([0.50,0.50])
},
"Reward + coverage bonus":{
0:np.array([0.35,0.65]),
1:np.array([1/3,1/3,1/3]),
2:np.array([0.90,0.10])
}
}

def entropy(p):
return -np.sum(p*np.log(p+eps))

def step(s,a):
if s==0:
return (1,0.0) if a==0 else (2,0.0)
if s==1:
return (1,0.1)
if s==2:
return (3,1.0) if a==0 else (4,0.0)
return (s,0.0)

def enumerate_policy(pi):
acc_return=0.0
acc_entropy=0.0
acc_decisions=0.0
acc_coverage=0.0
acc_useful=0.0
acc_success=0.0
stack=[(0,0,1.0,0.0,{0},0,0.0,0)]
while stack:
s,t,prob,G,visited,useful,Hsum,n_dec=stack.pop()
if t==H or s in terminal:
acc_return+=prob*G
acc_entropy+=prob*Hsum
acc_decisions+=prob*n_dec
acc_coverage+=prob*len(visited)
acc_useful+=prob*useful
acc_success+=prob*(1 if s==3 else 0)
continue
p=pi[s]
for a,pa in enumerate(p):
ns,r=step(s,a)
stack.append((ns,t+1,prob*pa,G+r,visited|{ns},
1 if (useful or (s==0 and a==1)) else 0,
Hsum+entropy(p),n_dec+1))
return acc_return,acc_entropy/acc_decisions,acc_coverage,acc_useful,acc_success

print("Policy, Return, Local action entropy, State coverage, Useful branch, Success")
for name,pi in policies.items():
vals=enumerate_policy(pi)
print(name+", "+", ".join(f"{v:.3f}" for v in vals))
